# *Caenorhabditis elegans* PAQR-2 and IGLR-2 Protect against Glucose Toxicity by Modulating Membrane Lipid Composition

**DOI:** 10.1371/journal.pgen.1005982

**Published:** 2016-04-15

**Authors:** Emma Svensk, Ranjan Devkota, Marcus Ståhlman, Parmida Ranji, Manish Rauthan, Fredrik Magnusson, Sofia Hammarsten, Maja Johansson, Jan Borén, Marc Pilon

**Affiliations:** 1 Department of Chemistry and Molecular Biology, University of Gothenburg, Gothenburg, Sweden; 2 Department of Molecular and Clinical Medicine/Wallenberg Laboratory, Institute of Medicine, University of Gothenburg, Gothenburg, Sweden; University of California San Francisco, UNITED STATES

## Abstract

In spite of the worldwide impact of diabetes on human health, the mechanisms behind glucose toxicity remain elusive. Here we show that *C*. *elegans* mutants lacking *paqr-2*, the worm homolog of the adiponectin receptors AdipoR1/2, or its newly identified functional partner *iglr-2*, are glucose intolerant and die in the presence of as little as 20 mM glucose. Using FRAP (Fluorescence Recovery After Photobleaching) on living worms, we found that cultivation in the presence of glucose causes a decrease in membrane fluidity in *paqr-2* and *iglr-2* mutants and that genetic suppressors of this sensitivity act to restore membrane fluidity by promoting fatty acid desaturation. The essential roles of *paqr-2* and *iglr-2* in the presence of glucose are completely independent from *daf-2* and *daf-16*, the *C*. *elegans* homologs of the insulin receptor and its downstream target FoxO, respectively. Using bimolecular fluorescence complementation, we also show that PAQR-2 and IGLR-2 interact on plasma membranes and thus may act together as a fluidity sensor that controls membrane lipid composition.

## Author Summary

Using the nematode *C*. *elegans* as a model, we show that glucose decreases the fluidity of cellular membranes, which suggests a novel mechanism for the glucose toxicity observed in diabetics. We also show that the membrane proteins PAQR-2 and IGLR-2 are located together on cellular membranes and regulate fluidity in the presence of glucose. This is an important finding because PAQR-2 is a worm homolog of AdipoR1 and AdipoR2, themselves important anti-diabetic proteins of which the cellular mechanism of action has been difficult to elucidate. Thus in one stroke our work may explain glucose toxicity, describes a cellular mechanism that counteracts it, helps clarify the anti-diabetic roles of AdipoR1/2, and identifies IGLR-2 as a PAQR-2 co-receptor.

## Introduction

Plasma glucose levels are maintained within a narrow range, and even moderately elevated levels are harmful [1]. In spite of the worldwide impact of diabetes on human health, the mechanisms behind glucose toxicity are not fully understood. Oxidative stress [2], advanced glycation end (AGE) products [3], lipotoxicity [4] and glucose flux through the hexosamine pathway [5] have been proposed as possible mechanisms, though none has received universal acceptance, suggesting a complex phenomenon. Indeed, and quoting from a relatively recent review: "… even as several studies have been carried out in the last 40 years, there is no unifying theory explaining the mechanism of the deleterious effects of glucose toxicity" [6]. We reasoned that glucose toxicity could perhaps be better understood by discovering how protective mechanisms operate. To this end, we studied *C*. *elegans* mutants lacking PAQR-2, a homolog of the human proteins AdipoR1 and AdipoR2 that have seven transmembrane domains, are unrelated to G- protein coupled receptors, and have anti-diabetic functions [7–9]. We previously showed that PAQR-2, like its mammalian homologs, regulates fatty acid (FA) metabolism: the *paqr-2* mutant shows an abnormal FA composition and is synthetic lethal with mutations in genes that promote FA turnover and desaturation, such as *sbp-1* and *nhr-49* (*C*. *elegans* homologs of SREBP and HNF4/PPARα [10–12]). Furthermore, the *paqr-2* mutant phenotypes can be suppressed genetically by mutations that promote FA desaturation [13,14].

Increasing the relative abundance of unsaturated FAs in biological membranes increases their fluidity [15–17], and this is likely the primary function of PAQR-2. Our previous studies suggest that PAQR-2 regulates membrane homeostasis and acts by promoting fatty acid desaturation in phospholipids during cold adaptation [13,14,18]. PAQR-2 therefore appears to act as a eukaryotic equivalent to DesK, a bacterial 5-transmembrane domain protein that senses membrane fluidity and activates a FA desaturase in response to excessive membrane rigidity [19–22]. The present study adds three main findings: 1) We identify IGLR-2 as an essential PAQR-2 partner for maintaining membrane homeostasis; 2) We show that supplying glucose to *C*. *elegans* causes a lethal decrease in membrane fluidity unless PAQR-2 and IGLR-2 are functional; and 3) We identify two ways to suppress the glucose toxicity in the *paqr-2* or *iglr-2* mutants, namely genetic suppression by mutations that cause increased FA desaturation, and chemical suppression with mild detergents. Previous studies have documented a reduced life span when *C*. *elegans* is cultivated in the presence of glucose, and implicated the insulin signalling pathway, e.g. DAF-2 and DAF-16, and FA desaturation, regulated by SBP-1 and MDT-15, as important mitigators of long-term glucose toxicity [23–25]. In contrast, the present study shows that PAQR-2 and IGLR-2 are essential to prevent acute glucose toxicity, and that they do not act via the insulin signalling pathway but rather by maintaining membrane homeostasis, at least in part via FA desaturation.

## Results

### The *paqr-2* mutant is glucose sensitive

**Fig 1:**
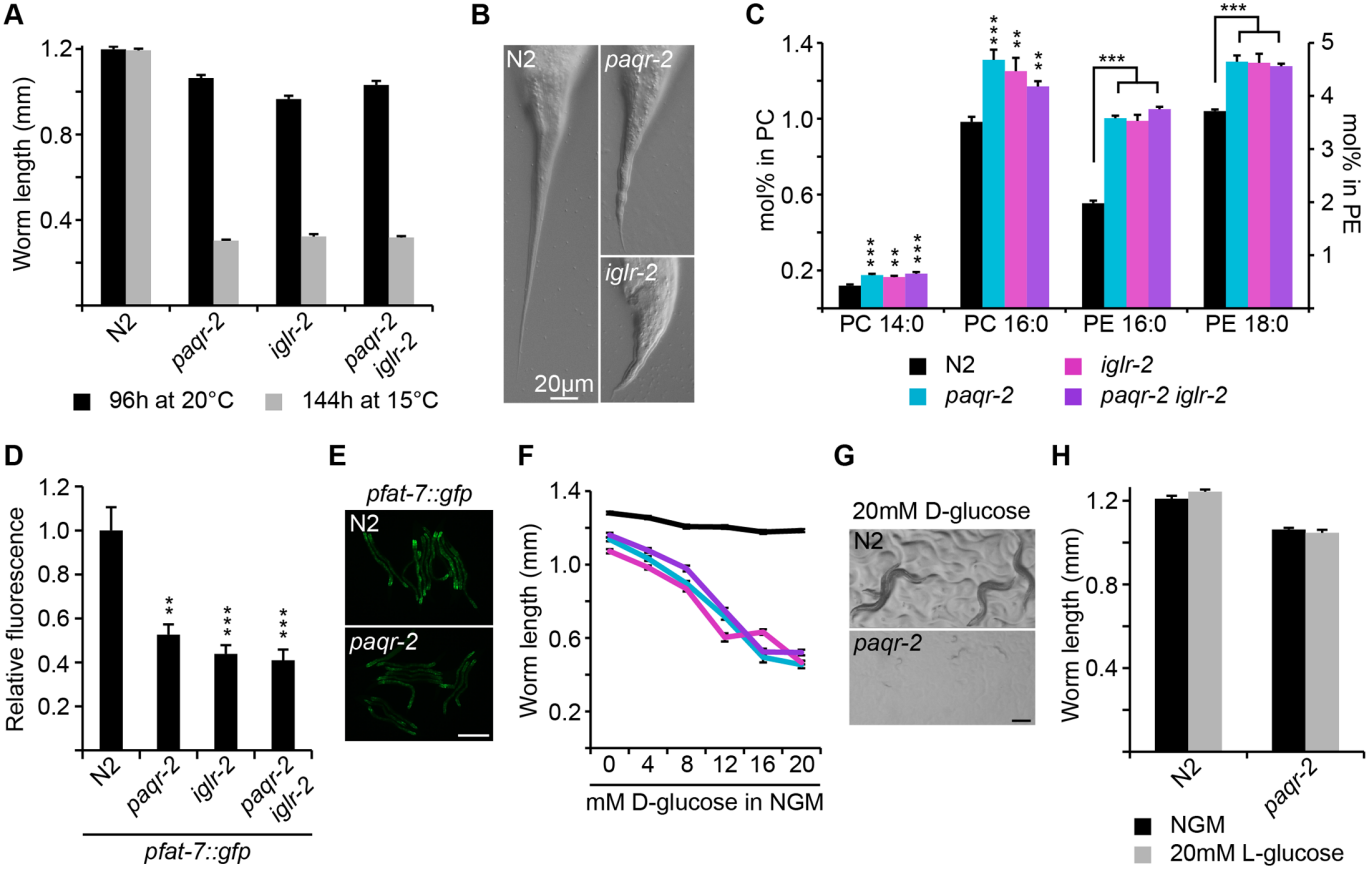
The *paqr-2* and *iglr-2* mutants share several phenotypes, including glucose intolerance. The *paqr-2(tm3410)* and *iglr-2(et34)* single and double mutants are unable to grow at 15°C **(A)**, have a withered tail tip defect **(B)**, have an excess of saturated fatty acids in phosphatidylcholine (PC) and phosphatidylethanolamine lipids (PE) **(C)**, reduced expression of the Δ9 desaturase *pfat-7*::*GFP* reporter (*rtIs30*) **(D-E)** and are glucose intolerant **(F-G)**. **(H)** L-glucose has no effect. ** *p* ≤ 0.01, *** p *≤* 0.001. Scale bar in **E** and **G** is 250 μm. Legend in **C** applies also to **F**.

The *C*. *elegans paqr-2* mutant has several distinct phenotypes [13,18]: it is cold sensitive (Fig 1A), has a withered tail tip (Fig 1B), and an excess of saturated fatty acids (SFAs; Fig 1C and S1 and S2 Tables) consistent with reduced expression of the Δ9 desaturase *fat-7* (Fig 1D and 1E). We also found that the *paqr-2* mutant is sugar intolerant, being especially sensitive to glucose: as little as 20 mM glucose is sufficient to cause complete growth arrest and lethality in *paqr-2* mutant larvae (Fig 1F and 1G and S1A Fig). More specifically, almost all *paqr-2* L1 larvae transferred to culture plates containing 20 mM glucose arrest as L1s and gradually die over the following few days. The effect of glucose is mostly reversible within the first 3 hours, but less so after longer exposures (S1B Fig). Note that Lee *et al*. independently observed that *paqr-2* mutants are sensitive to glucose, which they mention in the supplementary materials of a recent publication, though no quantification was then provided [25]. Importantly, the sensitivity is specific to D-glucose—L-glucose has no effect—which rules out direct chemical or biophysical mechanisms, such as glycation, and implicates a biological, enzymatic and metabolic response to glucose and other potent sugars (Fig 1H). The glucose sensitivity of the *paqr-2* mutant is a particularly thought-provoking phenotype because the homologs of PAQR-2, i.e. AdipoR1 and AdipoR2, are also metabolic regulators important for glucose tolerance in mammals [7,26]: conservation of this function in *C*. *elegans* therefore strengthens the case for using this organism as a model to elucidate the signalling pathway for these receptors.

### *paqr-2* and *iglr-2* mutants have identical phenotypes

**Fig 2:**
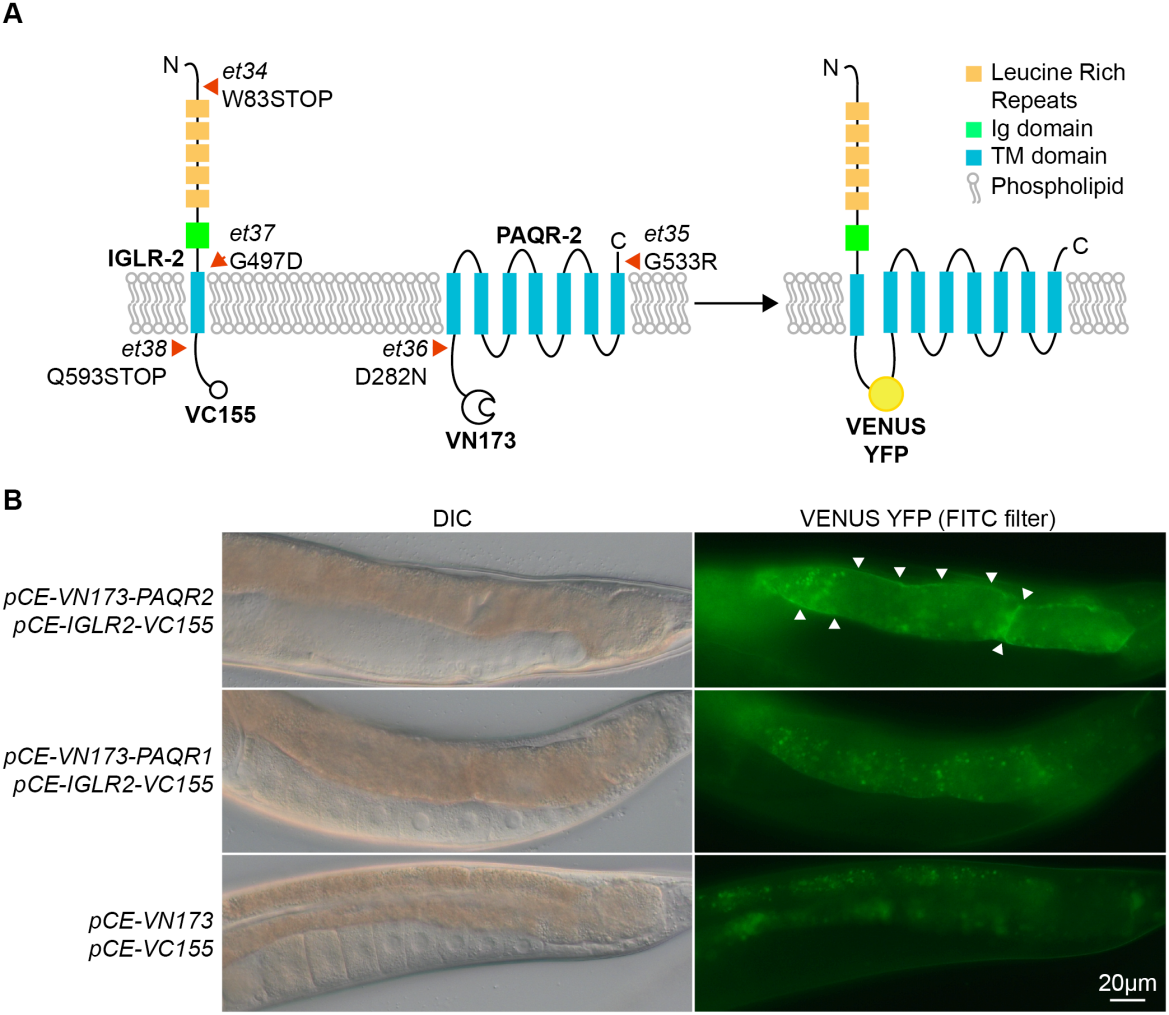
Novel alleles of PAQR-2 and IGLR-2, and interaction of IGLR-2 with PAQR-2. **(A)** Schematic structures of the IGLR-2 and PAQR-2 proteins, with novel mutations indicated by red arrowheads. The VC155 and VN173 fragments added to the C and N terminal ends of IGLR-2 and PAQR-2, respectively, allows reconstitution of a full and fluorescent VENUS YFP protein if the two proteins come into close proximity. **(B)** Result of the BiFC experiment showing that IGLR-2 and PAQR-2 contact each other on cellular membranes. The top two panels show a transgenic worm co-expressing the fusion proteins depicted in **(A)**; note the clear membrane-localized fluorescence indicative of IGLR-2 and PAQR-2 interaction. The middle two panels show a transgenic worm co-expressing the tagged IGLR-2 and a tagged PAQR-1 protein; note that only autofluorescent gut granules emit a signal, indicating that IGLR-2 and PAQR-1 do not interact with each other. The bottom two panels show a transgenic animal carrying the two empty vectors used in the BiFC experiments; note again that only autofluorescent gut granules emit a signal.

To identify novel components of the *paqr-2* pathway that are important for glucose tolerance, we sought mutants that have phenotypes identical to that of *paqr-2*, and which therefore may affect genes that act together with or downstream of PAQR-2. A forward genetics screen of over 80 000 mutagenized haploid genomes yielded 5 mutants with phenotypes identical to that of *paqr-2*: three were alleles of the gene *iglr-2* and two were novel alleles of *paqr-2* itself (Fig 2A and S2 Fig and Table 1). The *iglr-2* mutant alleles are remarkably similar to *paqr-2* in all assays tested: they exhibit the same cold sensitivity (Fig 1A and S1C Fig), tail tip defect (Fig 1B), excess of saturated fatty acid (Fig 1C and S1D Fig, S1 and S2 Tables), decreased *fat-7* expression (Fig 1D and S1E Fig), sugar intolerance (Fig 1F and S1A and S1F Fig), as well as reduced brood size (S1G Fig) and slow growth rate (S1H Fig). This striking similarity in phenotypes suggests that *iglr-2* and *paqr-2* act together as a complex or act in a simple direct sequence, one being downstream of the other. One prediction from these models is that the double mutant should exhibit the same phenotypes as the single mutants. This is indeed the case (Fig 1A, 1C, 1D and 1F, S1A, S1G and S1H Fig; and S1 and S2 Tables). In summary, genetic evidence suggests that *iglr-2* and *paqr-2* act in a mutually dependent way during cold adaptation, tail tip morphogenesis, regulation of fatty acid composition, and glucose tolerance.

**Table 1:** Description of the novel *paqr-*2 and *iglr-*2 alleles.

*Gene(allele)*	ORF name	ORF Length	Mutation
*paqr-2(et36)*	F58B6.3b	552 aa	*D(GAT)282N(AAT)*
*paqr-2(et35)*	F58B6.3b	552 aa	*G(GGA)533R(AGA)*
*iglr-2(et34)*	ZC262.3b	773 aa	*W(TGG)83STOP(TAG)*
*iglr-2(et37)*	ZC262.3b	773 aa	*G(GGT)497D(GAT)*
*iglr-2(et38)*	ZC262.3b	773 aa	*Q(CAA)593STOP(TAA)*

### IGLR-2 co-localizes with PAQR-2 on plasma membranes

IGLR-2 is predicted to consist of an intracellular C-terminal domain, a single transmembrane domain, and an extracellular part with an immunoglobulin (Ig)-like domain and several leucine rich repeats (LRRs) [27] (Fig 2A). The sequence and domain structure of IGLR-2 is related to that of nearly forty mammalian LRIG-type membrane proteins with a range of expression patterns and functions [28]. Some, such as LRIG1 regulate the activity and stability of growth factor receptors [29], while others, such as AMIGO, facilitate the clustering and activity of voltage-gated channels [30]. The three *iglr-2* alleles that we isolated are likely loss-of-function (*lof*) alleles: *et34* and *et38* introduce premature STOP codons while *et37* replaces a neutral glycine with the acidic amino acid aspartate within the transmembrane domain.

An IGLR-2::GFP translational reporter containing the same *iglr-2* gene and flanking sequence that efficiently rescues the mutant shows reproducible expression only on the plasma membranes of gonad sheath cells (S3 Fig). This is similar to the previously described expression profile of PAQR-2, which is also most readily observed in the gonad sheath cells, though low expression is also seen in some neurons of the head, ventral nerve cord and tail [13]. This suggests that PAQR-2 and IGLR-2 may have functions within the gonad sheath cells, a tissue that can regulate the metabolism and aging of other tissues [31,32]. We used Bimolecular Fluorescence Complementation analysis (BiFC) to test whether PAQR-2 and IGLR-2 actually interacts with each other. BiFC is a powerful method to visualize protein interactions in vivo that relies on fusing two separate portions of the Venus yellow fluorescent protein to each putative protein partner: physical interaction between the partners brings the complementary fragments of the fluorescent protein in close proximity, allowing its assembly and fluorescence [33,34]. BiFC shows that PAQR-2 and IGLR-2 interact on cell membranes (Fig 2B). The BiFC interaction between PAQR-2 and IGLR-2 is specific: BiFC revealed no interaction between IGLR-2 and PAQR-1, which we used as a control because of its structural similarity to PAQR-2 and which produced no fluorescence besides the endogenous autofluorescent gut granules present in transgenic worms containing empty vectors (Fig 2B). We conclude that PAQR-2 and IGLR-2 can form a complex on plasma membranes, and that this likely explains the genetic evidence for mutual dependence.

One hypothesis to explain the mutual dependency of PAQR-2 and IGLR-2 is that one requires the other for membrane localization or stability. This was tested by expressing the translational reporter for *paqr-2* in the *iglr-2* mutant, and vice versa. Expression of the *pIGLR-2*::*GFP* reporter is the same in wild-type and *paqr-2* mutant worms (S3A Fig). IGLR-2 therefore does not depend on PAQR-2 for its expression or localization to the gonad sheath cell membranes. In contrast, there is a dramatic reduction in the levels and frequency of expression of the *pPAQR-2*::*GFP* reporter in the *iglr-2* mutant background (S3A Fig). This indicates that IGLR-2 is important for PAQR-2 localization in gonad sheath cell membranes. IGLR-2 may therefore facilitate expression, processing, transport or stability of PAQR-2.

One of the novel *paqr-2* mutant alleles, *paqr-2(et36)* is a point mutation within the cytoplasmic domain, 30 amino acids from the first transmembrane domain, a region proposed to regulate the membrane localization of the mammalian homologs AdipoR1 and AdipoR2 [35–37]. Using a GFP translational reporter and the BiFC assay, we found that *paqr-2(et36)* has the same membrane localization and interaction with IGLR-2 as the wild-type allele, suggesting that *et36* does not interfere with either process (S3B Fig).

### Mutations affecting fatty acid metabolism can suppress the glucose sensitivity

*paqr-2* and *iglr-2* are essential for *C*. *elegans* survival in the presence of glucose. To pinpoint the mechanisms behind this glucose sensitivity, we screened a collection of mutations that we previously identified as suppressors of the *paqr-2* cold sensitivity and tail tip defect [18]. These suppressors fall into three classes, and each were tested for their ability to suppress the glucose sensitivity in single and double *paqr-2* and *iglr-2* mutants.

**Fig 3:**
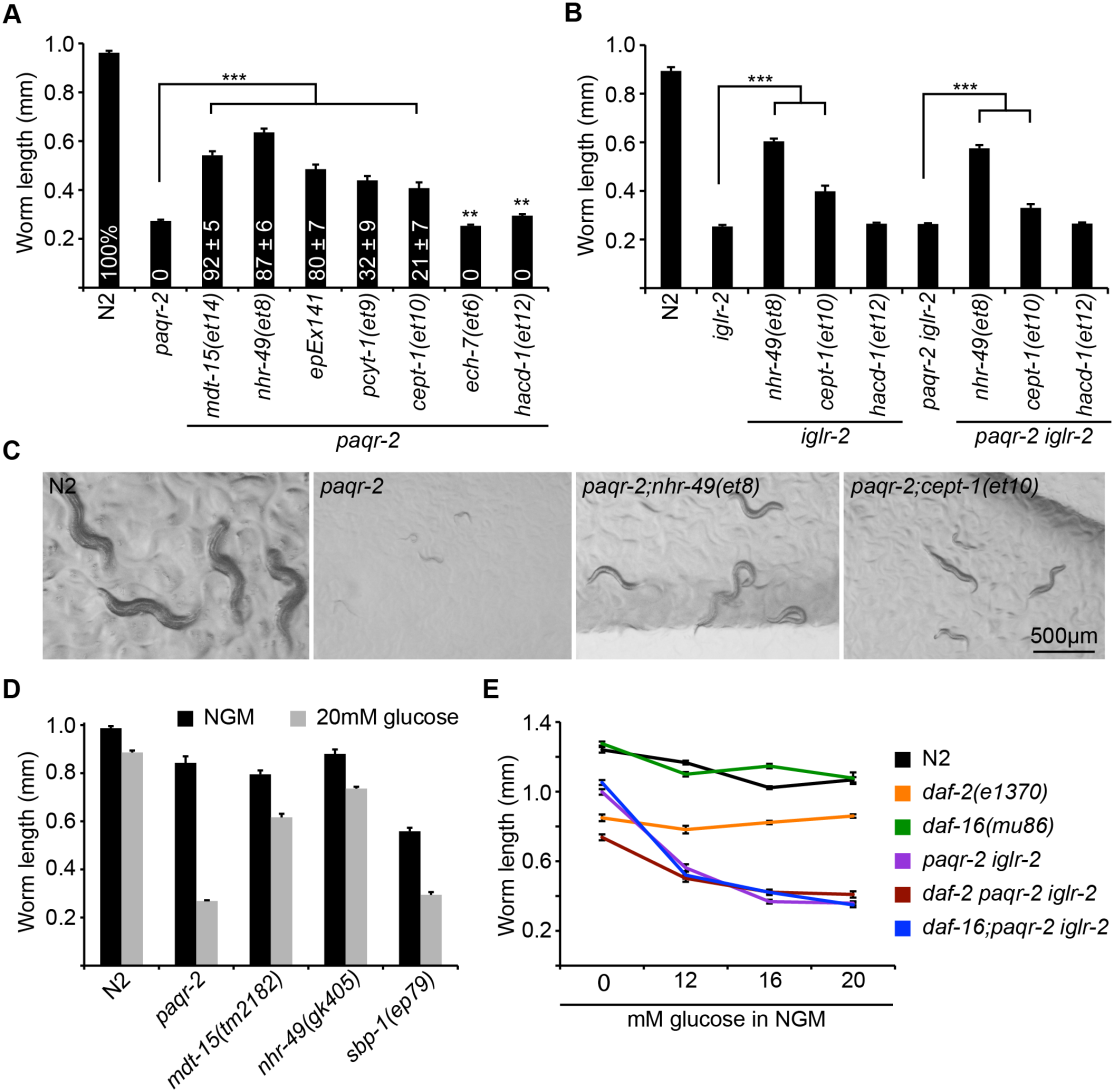
Genetic suppression of the *iglr-2* and *paqr-2* glucose sensitivity, and independence from the *daf-2/daf-16* pathway. **(A-B)** Glucose sensitivity in the *paqr-*2 and *iglr-2* single and double mutants is suppressed by *gof* mutations in *mdt-15* or *nhr-49*, an overexpression *sbp1* transgene (*epEx141*), and *lof* alleles of *pcyt-1* or *cept-1*, but not *lof* alleles of *ech-7* or *hacd-1*. The values within the bars in **(A)** indicate the fraction of animals that grow into fertile adults (n > 100). Note that the effect of the *sbp-1* transgene is probably underestimated for two reasons: it is an extrachromosomal array with variable mosaicism and it carries *rol-6* as a marker, which by itself causes the *paqr-2* mutant adults to be slightly shorter (0.995 ± 0.058 mm without *rol-6* vs 0.895 ± 0.061 mm with *rol-6*). **(C)** Representative images of worms of the indicated genotypes after 72 h of cultivation on 20 mM glucose. **(D)** The *lof* alleles of *mdt-15* and *sbp-1* show a clear glucose sensitivity, with *mdt-15(tm2182)* producing nearly 100% nonviable eggs (S4E Fig) and *sbp-1(ep79)* exhibiting a severely thwarted growth on 20 mM glucose. The *nhr-49(gk405) lof* allele is only slightly glucose sensitive. **(E)** The *daf-2(e1370)* and *daf-16(mu86)* mutants are not glucose sensitive and do not alter the glucose sensitivity of the *paqr-2 iglr-2* double mutant, suggesting that these genes act independently of *daf-2* and *daf-16*. *** p *≤* 0.001.

The first class of suppressors are those with loss-of-function (*lof*) mutations in genes encoding enzymes of the fatty acid beta-oxidation pathway (*ech-7(et6)* and *hacd-1(et12)*) [38]. These had no effect on the glucose sensitivity of the *paqr-2* mutant (Fig 3A). Mutations in *acdh-11*, which acts upstream of *ech-7* and *hacd-1* during FA beta-oxidation, were recently shown to also act as *paqr-2* suppressors [39]. The *acdh-11(gk753061) lof* allele did not suppress the glucose sensitivity of the *paqr-2* mutant (S4B Fig), though it did rescue the cold adaptation and tail tip defect (S4C Fig). *hacd-1(et12)*, chosen as a representative of this class, also had no effect on the glucose sensitivity of the *iglr-2* single or *paqr-2 iglr-2* double mutants (Fig 3B), but could readily suppress the cold sensitivity of the *paqr-2* and *iglr-2* single or double mutants (S4D Fig). We conclude that mutations in the FA beta-oxidation pathway are not suppressors of the glucose sensitivity in the *paqr-2* or *iglr-2* mutants, though they can suppress other phenotypes.

The second class of mutations examined are *lof* alleles of genes in the phosphatidylcholine/ethanolamine biosynthesis pathway (*sams-1(ok2946)*, *pcyt-1(et9)* and *cept-1(et10 and 11)*), which are thought to act by indirectly promoting *sbp-1* activity [12]. Of these, only *cept-1* and *pcyt-1* partially suppressed the glucose sensitivity of the *paqr-2* mutant (Fig 3A and 3C and S4A Fig). *cept-1(et10)*, chosen as a representative of this class, also partially suppressed the glucose sensitivity of the *iglr-2* single or *paqr-2 iglr-2* double mutants (Fig 3B), and could readily suppress the cold sensitivity of the *paqr-2* and *iglr-2* single or double mutants (S4D Fig). We conclude that mutations in the phosphatidylcholine/ethanolamine biosynthesis pathway are partial suppressors of the glucose sensitivity in the *paqr-2* or *iglr-2* mutants, and strong suppressors of their cold adaptation defect.

Finally, the third class of *paqr-2* suppressors are gain-of-function (*gof*) mutations in transcriptional regulators of metabolism, notably acting as activators of Δ9 desaturases and causing a significant increase in the proportion of unsaturated fatty acids (UFAs) in membrane phospholipids and triglycerides (*nhr-49(et7*, *et8* and *et13)*, *mdt-15(et14)*, and the *sbp-1* overexpression transgene *epEx141*) [10,11,18,40–42]. These mutations and the transgene conferred increased resistance to the *paqr-2* mutant (Fig 3A and 3C and S4A Fig), allowing growth and reproduction in the presence of 20 mM glucose. *nhr-49(et8)* chosen as a representative of this class, also strongly suppressed the glucose sensitivity of the *iglr-2* single or *paqr-2 iglr-2* double mutants (Fig 3B), and could readily suppress the cold sensitivity of the *paqr-2* and *iglr-2* single or double mutants (S4D Fig). Interestingly, *lof* mutations in *mdt-15* and *sbp-1* also cause a glucose tolerance defect, though not as severe as in the *paqr-2* or *iglr-2* mutants (Fig 3D and S4E Fig), which suggests that *mdt-15* and/or *sbp-1* are among the downstream targets of *paqr-2* and *iglr-2* in response to glucose exposure.

We conclude from these genetic interaction studies of *paqr-2* suppressors that regulation of fatty acid metabolism by PAQR-2 and IGLR-2, and specifically upregulation of Δ9 desaturases that are key regulators of membrane composition and turnover [38,43], is a particularly important aspect of glucose adaptation in *C*. *elegans*.

### *paqr-2* and *iglr-2* act separately from the insulin signalling pathway

The insulin signalling pathway is an important part of the nutrient/glucose response in most organisms, and therefore an obvious candidate for contributing to the glucose sensitivity observed in the *paqr-2* and *iglr-2* mutants. DAF-2 is the *C*. *elegans* homolog of the insulin receptor and an important regulator of metabolism: DAF-2 signalling promotes growth and reproduction when food is available, while lack of DAF-2 signalling during starvation allows activation of the forkhead transcription factor DAF-16, which promotes “dauer” development hence fat storage, stress resistance and longevity [44,45]. Suppression of DAF-16 accounts for the reduced lifespan of wild type worms grown on 20 mM glucose [23]. Importantly, the *daf-2* and *daf-16* mutants showed no glucose sensitivity in our short term, acute toxicity assay, nor did they enhance the sensitivity of the *paqr-2 iglr-2* double mutant (Fig 3E). The roles of PAQR-2 and IGLR-2 during glucose exposure are therefore unrelated to insulin signalling and represent an entirely new pathway essential for survival in the presence of glucose.

### Glucose causes decreased membrane fluidity

**Fig 4:**
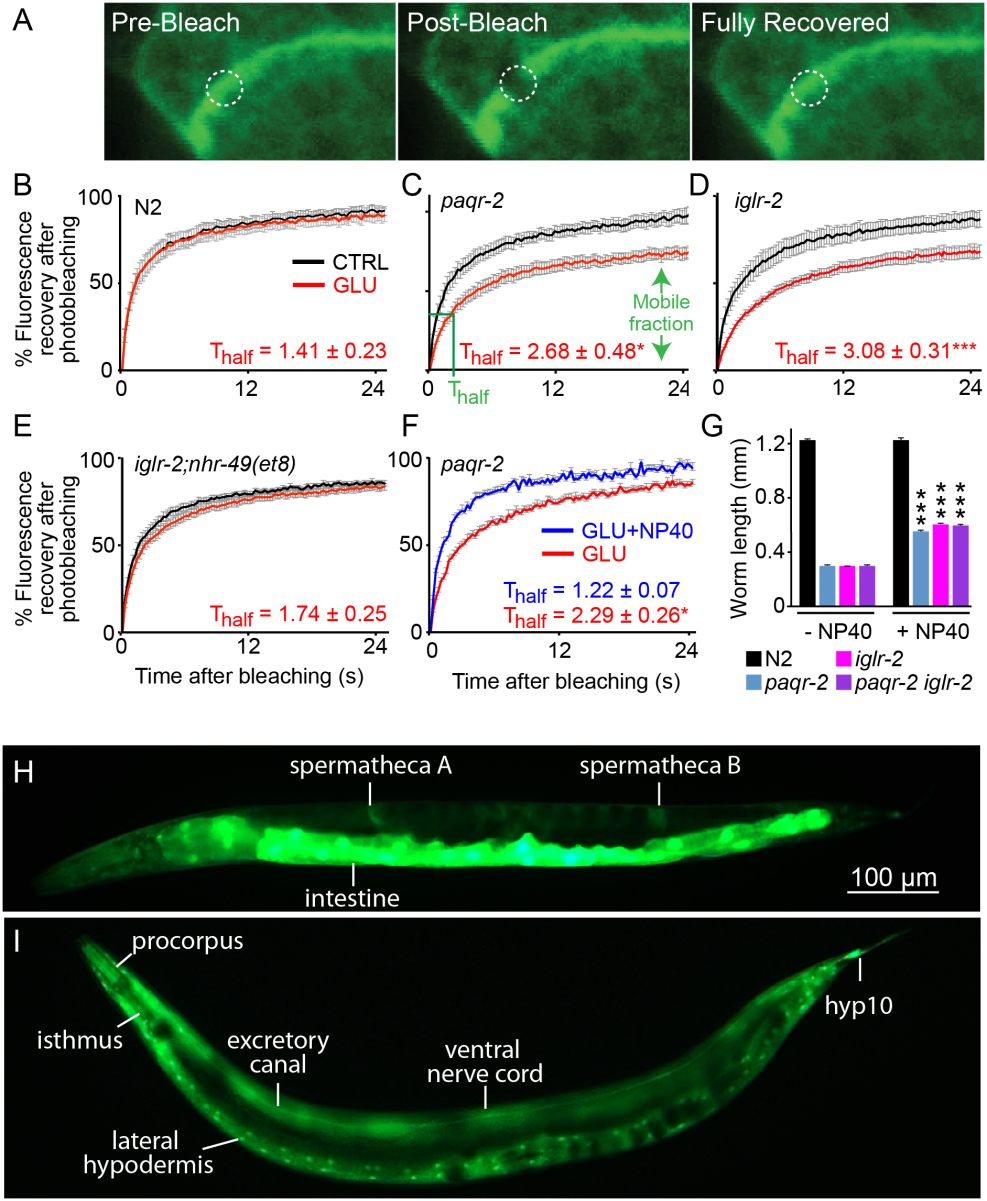
The *paqr-2* and *iglr-2* mutants are unable to maintain membrane fluidity in presence of glucose. **(A)** Example of a photobleaching experiment on a worm transgenic for the *pGLO-1P*::*GFP-CAAX* construct, which expresses a prenylated and thus membrane associated GFP in intestinal cells. The GFP signal was fully recovered within 30 seconds after photobleaching. **(B-F)** Curves of fluorescence recovery after photobleaching showing that membrane fluidity in wild-type worms is unaffected by overnight cultivation on 20 mM glucose **(B)**, severely reduced in *paqr-2* and *iglr-2* mutants grown on glucose both in terms of a slower diffusion rate, approximated by the half-time of recovery (t_half_ value) and a reduced mobile fraction, which are indicated for *paqr-2* on glucose **(C-D)**, but unaffected in *iglr-2;nhr-49(et8)* mutants grown on glucose **(E)**. **(F)** Inclusion of 0.1% NP-40 rescues the membrane fluidity in *paqr-2* mutant worms grown in the presence of 20 mM glucose. The curves show the average fluorescence over time, and error bars represent the sem; n>8. **(G)** Inclusion of 0.1% NP-40, a non-ionic detergent, partially rescues the *paqr-2* and *iglr-2* single and double mutants from the toxic effects of glucose. **(H-I)** Mosaic analysis of *iglr-2; Ex[SUR-5GFP(NLS) pIGLR-2]* transgenic worms that grew to adulthood on 20 mM glucose. The worm in **(H)** carries the extrachromosomal array in intestinal cells, spermatheca and many others cells. The worm in **(I)** carries the extrachromosomal array in hypodermal cells, pharyngeal procorpus and isthmus and many other cells (some are out of focus), but not in the intestine. * *p* ≤ 0.05, ** *p* ≤ 0.01, *** p *≤* 0.001.

As noted earlier, the best suppressors of the glucose sensitivity in the *paqr-2* and *iglr-2* mutants are those that most directly activate expression of Δ9 desaturases, and hence should mediate the largest increase in membrane fluidity [46]. This is further supported by the observation that the *paqr-2* and *iglr-2* mutants have an excess of saturated fatty acids in their membranes, as noted earlier (Fig 1C and S1 and S2 Tables). We previously hypothesized that the primary function of PAQR-2 during cold adaptation is to maintain membrane fluidity by upregulating Δ9 desaturases and thus increase the proportion of unsaturated phospholipids in membranes [18]. Could the same function explain glucose sensitivity in the *paqr-2* and *iglr-2* mutants? In other words, could the availability of glucose promote the saturation of membrane lipids, hence membrane rigidity, to an extent that is incompatible with growth and survival unless compensatory desaturation, regulated by *paqr-2* and *iglr-2*, occurs? To begin addressing this experimentally, we directly and quantitatively measured membrane fluidity *in vivo* using fluorescence recovery after photobleaching (FRAP). In FRAP experiments, fluorescent molecules, such as GFP, are photobleached in a small area of the cell using a high-powered laser, and subsequent diffusion of non-bleached fluorescent molecules into the bleached area leads to recovery of fluorescence, which is recorded and quantified (Fig 4A) [47]. Using FRAP on transgenic worms expressing a prenylated GFP in intestinal membranes, we first confirmed that at the permissive temperature (20°C), the *paqr-2* and *iglr-2* mutants are indistinguishable from wild type, whereas at 15°C these mutant worms exhibit significantly reduced membrane fluidity (S5A–S5C Fig). This provides strong experimental support for the hypothesis that *paqr-2* and *iglr-2* are regulators of membrane fluidity. Next, we examined membrane fluidity in the presence of glucose. Remarkably, the *paqr-2* and *iglr-2* mutant worms showed a clear decrease in membrane fluidity when cultivated in the presence of 20 mM glucose, which had no effect on wild-type worms (Fig 4B–4D). Furthermore, the drop in fluidity was suppressed when the *nhr-49(et8) gof* allele was combined with the *iglr-2* mutation; this is consistent with the *nhr-49(et8)* allele acting as an inducer of the Δ9 desaturases with the net effect being to normalize membrane fluidity (Fig 4E). The increased membrane rigidity is likely due to changes in membrane composition: lipidomics profiling revealed a strong increase in the proportion of saturated FAs in phospholipids, especially among PE species, which are the most abundant phospholipids in *C*. *elegans* [48], when the mutants are cultivated in the presence of glucose (S5D Fig). Finally, and consistent with the hypothesis that glucose toxicity results from altered membrane fluidity, small amounts of a non-ionic detergent, which measurably increases membrane fluidity (Fig 4F) [49], partially suppressed the glucose sensitivity of the *paqr-2* and *iglr-2* mutants (Fig 4G).

### IGLR-2 acts in a cell non-autonomous manner

We showed earlier that PAQR-2 and IGLR-2 are most reproducibly expressed in the gonad sheath cells, where IGLR-2 is important for PAQR-2 membrane localization (S3 Fig)[13]. However, the FRAP results show that PAQR-2 and IGLR-2 regulate membrane fluidity in the intestine when cultivated in the cold or in the presence of glucose (Fig 4B–4D and S5 Fig), and the lipidomics analysis of entire worms shows significant changes in the phospholipid composition of the mutant worms, especially when cultivated in the presence of glucose (S5D Fig). Taken together, these results suggest that PAQR-2 and IGLR-2 may act cell non-autonomously to systemically regulate FA metabolism and membrane properties. We tested this hypothesis by performing a mosaic analysis. In *C*. *elegans*, transgenes are typically retained as multicopy extrachromosomal arrays that are not always segregated to both daughter cells during cell division, resulting in genetic mosaics [50]. Mosaic analysis has the merit that it makes no assumption about the specificity of putative tissue-specific promoters where weak expression in some tissues may go undetected. Similarly, it also does not rely on the visible expression pattern of a gene of interest based on reporter constructs that may also go undetected in weakly expressing tissues.

For our study, we used the SUR-5GFP(NLS) reporter previously developed for this purpose [51]. This reporter is expressed in nearly all nuclei, as well as weakly in the cytoplasm of adult cells, being especially strong in intestine (which can obscure expression in other cells). We scored *iglr-*2 mutant worms that grew from L1 to adults on 20 mM glucose while carrying an extrachromosomal array harboring SUR-5-GFP(NLS) together with a rescuing *iglr-2* transgene. An initial survey of 150 such transgenic worms identified 6 that did not carry the transgene in intestinal cells (Fig 4H and 4I). This demonstrates that IGLR-2 is not required in intestinal cells to permit resistance to glucose, but must instead have its essential function in some other tissue. Close inspection of 16 worms that grew into adults on 20 mM glucose while lacking intestinal expression revealed that *iglr-2* is also definitely not required in the MS lineage, which produces the gonad sheath cells, nor in the C and D lineages, which produce mostly body wall muscles (Table 2). In contrast, descendants of ABa and ABp, and more specifically the hypodermis, were always positive for the transgene in glucose-tolerant worms. This strongly suggests that the hypodermis, or perhaps some neuron(s) of the AB lineage, is the essential site of *iglr-2* activity for glucose tolerance. Note that this result does not exclude the possibility that PAQR-2 and IGLR-2 may also have important functions in the gonad sheath cells, though such functions would clearly not be essential for glucose tolerance.

**Table 2:** Result of the mosaic analysis of *iglr-2;Ex[SUR-5*::*GFP(NLS) pIGLR-2]* worms selected for growth to adulthood on 20 mM glucose. (+/-) indicates that the majority of the tissue was positive, while (-/+) indicates that only a minority was positive for GFP.

Cells/tissue (lineage) scored in each worm	1	2	3	4	5	6	7	8	9	10	11	12	13	14	15	16
Intestine (E)	-	-	-	-	-	-	-	-	-	-	-	-	-	-	-	-
Spermatheca A (MSpppapp)	-	-	-	-	-	+	-	-	-	-	-	-	+	-	-	-
Spermatheca P (MSappaap)	-	-	-	-	-	+	-	-	-	-	-	-	+	-	-	-
Gonad sheath A (Mspppapp)	-	-	-	-	-	+	-	-	-	-	-	-	+	-	-	-
Gonad sheath P (Msappaap)	-	-	-	-	-	+	-	-	-	-	-	-	+	-	-	-
Lateral hypodermis (ABa, ABp, C)	+	+	+	+	+	+	+	+	+	+	+	+	+	+	+	+
Ventral hypodermal ridge (ABp)	+	+	+	+	+	+	+	+	+	+	+	-	+	+	+	+
Excretory canal (ABplpa)	+	-	-	+	+	+	+	+	-	-	+	-	+	+	+	+
Procorpus (4 ABa/2 MS)	+/-	+/-	+/-	+/-	+/-	+	+/-	+/-	+/-	+/-	+	-	+	+/-	+/-	+/-
Metacorpus (5 MS/1 ABar)	-	-	-	-	-	+	-	-	-	-	+	-	+	-	-	-
Isthmus (4 MS/2 ABaraa)	-/+	-/+	-/+	-/+	-/+	+	-/+	-/+	-/+	-/+	+	-	+	-/+	-/+	-/+
Posterior bulb (MS)	-	-	-	-	-	+	-	-	-	-	+	-	+	-	-	-
Hyp 10 (AB)	+	+	-	+	+	-	+	+	+	+	-	-	+	+	-	+
Body wall muscles Head (MS)	+	-	+	+	-	-	-	-	-	-	+	-	+	-	-	-
Body wall muscles Mid (D)	-	-	-	-	-	-	-	-	-	-	+	-	+	-	-	-
Body wall muscles Posterior (C)	+	-	-	+	-	-	-	-	-	-	-	-	-	-	-	-
VC2-6 neurons (ABp)	-	+	-	-	+	-	+	+	-	+	-	-	-	-	-	-
Head ganglion neurons (mostly ABp)	-/+	+/-	-/+	-/+	+/-	-/+	-/+	-/+	+/-	+/-	-/+	-/+	-/+	-/+	-/+	-/+
Ventral nerve cord neurons (mostly ABp)	-/+	+/-	-/+	-/+	+/-	-/+	-/+	-/+	+/-	+/-	-/+	-/+	-/+	-/+	-/+	-/+
---------------------------------------------------------- Likely missing from:	E, D, MS	P1	E, C, D, most MS	E, D, most MS	P1	E, C, D	P1	P1	P1	P1	E, C	P1, most ABa?	E, C	P1	P1	P1
Present in:	ABa, ABp, C	ABa, ABp	ABa, ABp	ABa, ABp, C	ABa, ABp	ABa, ABp MSa, MSp,	ABa, ABp	ABa, ABp	ABa, ABp	ABa, ABp	ABa, ABp, D, MS	ABp	ABa, ABp, D, MS	ABa, ABp	ABa, ABp	ABa, ABp

## Discussion

**Fig 5:**
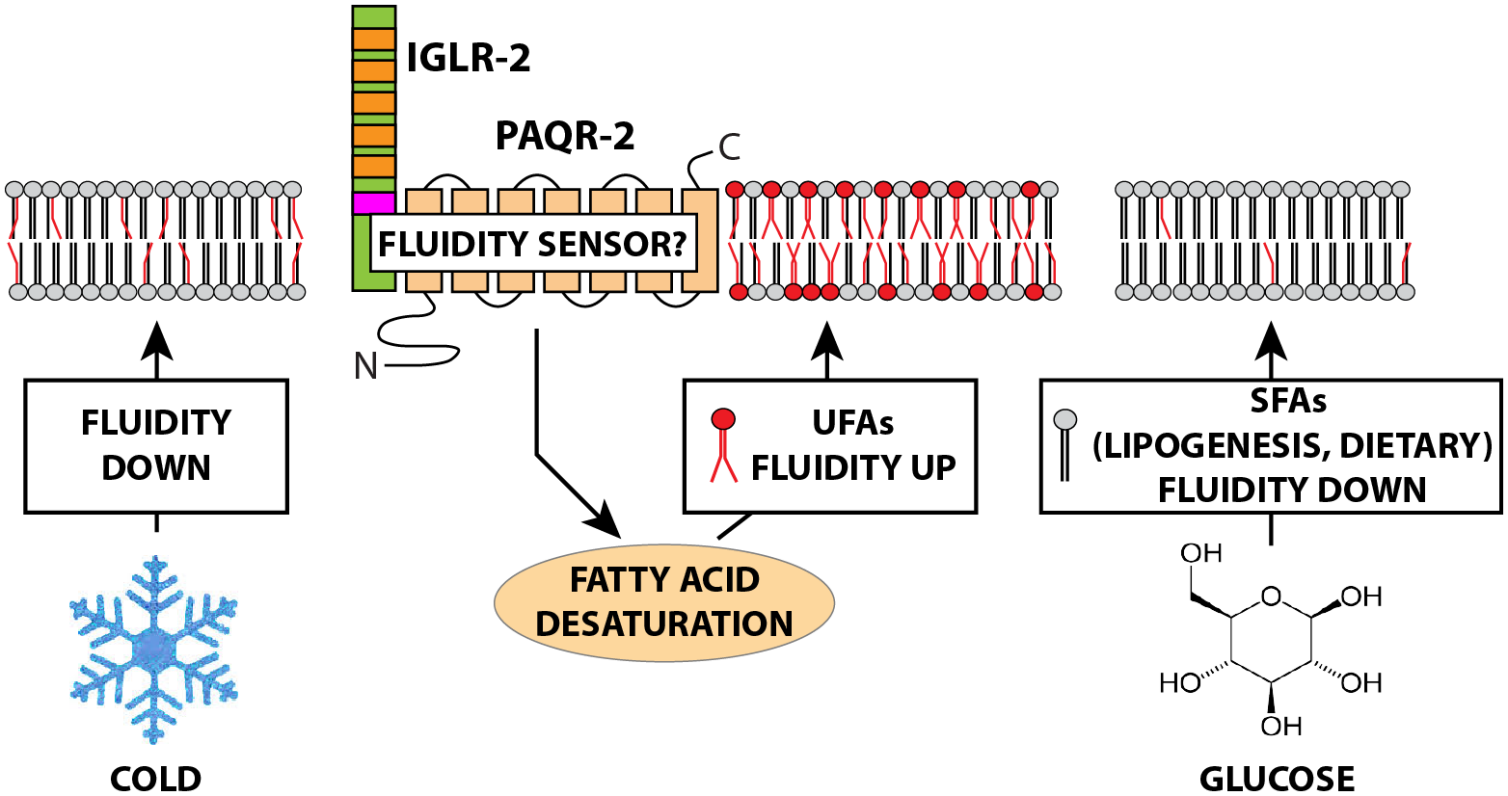
Model of membrane fluidity regulation by PAQR-2 and IGLR-2. The model proposes that PAQR-2 and IGLR-2 act as sensors that are activated by low membrane fluidity and act on downstream effectors to restore fluidity by promoting changes in fatty acid metabolism, including promoting the activity of Δ9 desaturases. Cold or availability of glucose tend to decrease membrane fluidity, which explains the sensitivity phenotypes of the *paqr-2* and *iglr-2* mutants. The effect of low temperature on membrane fluidity is purely biophysical, while the effect of glucose is indirect and may be attributed to lipogenesis whereby glucose is converted into saturated fatty acids or to an effect on the dietary FA in the *E*. *coli* used as food source. In the presence of glucose the relative abundance of saturated fatty acids increases within the available pool, leading to an increase in membrane rigidity as phospholipid turnover occurs.

We previously proposed that *C*. *elegans* PAQR-2 regulates membrane homeostasis by promoting fatty acid desaturation in phospholipids, for example by increasing membrane fluidity during cold adaptation [13,14,18]. The present study adds three main findings: 1) We identify IGLR-2 as an essential PAQR-2 partner for maintaining membrane homeostasis and show that these proteins likely act in the hypodermis and/or gonad sheath cells; 2) We show quantitatively that supplying glucose to *C*. *elegans* causes an excess of SFAs in phospholipids and a lethal decrease in membrane fluidity unless PAQR-2 and IGLR-2 are functional; and 3) We identified two ways to suppress the glucose toxicity in the *paqr-2* or *iglr-2* mutants: genetic suppression by mutations that cause increased fatty acid desaturation, and chemical suppression with detergents. The availability of glucose, being a superior energy source, causes important changes in FA metabolism [23,24,52,53], reducing the need for beta-oxidation and increasing lipogenesis whereby glucose is converted to SFAs. The presence of glucose in the culture plates may also lead to an increase in dietary SFAs since the *E*. *coli* that is provided as food source can also metabolize glucose into fatty acids [54]. Given that membrane lipids are constantly being turned over [43], the result is an increase in the relative abundance of saturated and rigidity-prone fatty acids in cellular membranes. Unless coordinated with a compensatory activity of desaturases, membrane fluidity will suffer. Although the ability to adjust membrane fluidity in response to environmental factors is particularly important in poikilotherms, the impact of diet or metabolic changes on membrane lipid composition makes the need to monitor and modify fluidity universal [55,56]. We propose a model where PAQR-2 and IGLR-2, and presumably their mammalian homologs, constitute parts of such a system, and as membrane proteins they are excellent candidates for acting as a fluidity sensor (Fig 5). Speculatively, formation of a PAQR-2/IGLR-2 complex may be facilitated by low membrane fluidity, leading to activation and downstream signalling to effectors such as NHR-49, MDT-15 or SBP-1 that promote fatty acid desaturation. The nature of this putative signal is unknown, but PAQR-2 and its mammalian homologs, AdipoR1/2, is thought to contain a ceramidase or phospholipase-like domain that could generate a lipid acting as ligand for downstream targets [8,39,57,58].

### IGLR-2 and PAQR-2 regulate FA desaturation during glucose adaptation

We previously performed a *paqr-2* suppressor screen that led to the isolation of 9 novel mutant alleles affecting 6 different genes that could suppress the cold sensitivity and tail tip defect in the *paqr-2* mutant [18]. Among these, the most potent suppressors were three *gof* alleles in *nhr-49*, and one *gof* allele of *mdt-15*. Subsequent studies also revealed that a *sbp-1* multi-copy transgene, which likely also acts as a *gof* allele, is also a potent *paqr-2* suppressor. The common denominator that may functionally link *nhr-49*, *sbp-1* and *mdt-15* with respect to membrane homeostasis is their effect on fatty acid metabolism, namely that all three activate the Δ9 desaturase genes in *C*. *elegans*. These same suppressors were also the most potent suppressors of the glucose sensitivity in the *paqr-2* and *iglr-2* single or double mutants, suggesting an essential requirement for desaturase activity when worms are cultivated in the presence of glucose. Lee *et al*. made a similar observation: they showed that addition of glucose to the culture plates causes increased saturated fat content in wild type *C*. *elegans* and that SBP-1 and MDT-15 protect against glucose toxicity on lifespan by promoting fatty acid desaturation [25]. Additionally, they implicated excess production of dihydroxyacetonephosphate, an intermediate metabolite in glycolysis, as a toxic molecule that causes short life span on a glucose-rich diet. It is important to note that Lee *et al*. studied the effects of rather high concentrations of glucose (2%, or 111 mM) on the life span of wild-type or RNAi-treated worms, suggesting a more long-term toxicity in these conditions. In contrast, the *paqr-2* or *iglr-2* single or double mutants that were the focus of our study suffer much more rapidly from cultivation in the presence of smaller amounts of glucose, becoming developmentally arrested as L1s when cultivated in the presence of 20 mM glucose. This indicates a very direct and essential function, e.g. membrane homeostasis, for PAQR-2 and IGLR-2 upon cultivation in the presence of glucose, and that regulation of SBP-1 and MDT-15 may represent only a subset of the targets that are regulated by PAQR-2 and IGLR-2.

### IGLR-2 is related to LRIG proteins

Our screen to isolate mutants phenotypically similar to *paqr-2* yielded five alleles affecting only two genes, namely *iglr-2* and *paqr-2*. This suggests that it will be difficult to isolate more components of the *paqr-2* pathway using this approach. IGLR-2 is related to the mammalian LRIG proteins, and is the first *C*. *elegans* member of that protein family to be characterized. In mammals, LRIG proteins vary in their expression patterns and functions [28]. Some, such as LRIG1, 2 and 3 act as regulators of receptor tyrosine kinases, while others, such as the NLRRs, act as adhesion or signalling molecules [59,60]. Structurally, IGLR-2 is most similar to the mammalian AMIGO: both proteins have relatively few LRRs (5 and 7, respectively), a single Ig domain and a relatively short cytoplasmic domain. Intriguingly, AMIGO acts as an anchor point around which several Kv2.1 channel complexes can cluster, which increases their activity [30]. IGLR-2 could play a similar role in facilitating PAQR-2 clustering and stability to promote its activity, with the additional refinement that the interaction may also be regulated by membrane fluidity.

### PAQR-2 and IGLR-2 may be functionally analogous to the bacterial DesKR system

Dynamic regulation of membrane composition may be particularly important in an organism such as *C*. *elegans* where the majority of the membrane fatty acids are replaced within 24 hours [43]. While membrane turnover is slower in mammals [61–67], it does occur constantly and, clearly, mechanisms must exist to monitor membrane properties and correspondingly adjust their composition for example in response to various diets. Virtually all cellular processes are in some fashion influenced by membranes, and regulating their composition is the primary mechanism to maintain optimal membrane properties [46,68–70]. Perhaps the best understood sensor and regulator of membrane properties is the bacterial DesKR two-component system, which includes the histidine kinase DesK, a membrane protein with five transmembrane domains and a cytoplasmic catalytic domain containing the dimerization, histidine phosphotransferase and ATP-binding domains [19–21]. DesK is a bifunctional enzyme: it acts as a phosphatase when unstimulated and as a kinase when stimulated by a reduction in membrane fluidity, thus regulating the activity of its cognate response regulator, DesR, itself a transcriptional regulator of Δ5-Des, a Δ5-desaturase gene. The activity of DesK is regulated by a conformational change: fluidity sensing involves a built-in structural instability near the N terminus of the first transmembrane domain that is buried in the lipid phase at low temperature but partially “buoy” to the aqueous phase at higher temperature with the thinning of the membrane, promoting the required conformational change [22]. Perhaps an aspect of PAQR-2 and/or IGLR-2 conformation is similarly regulated by membrane properties during cold adaptation or growth in the presence of glucose. Failure to correct membrane composition under these conditions, as in the *paqr-2* or *iglr-2* mutants, results in intolerable membrane rigidity.

### PAQR-2 and IGLR-2 can act cell non-autonomously

PAQR-2 and IGLR-2 likely act cell non-autonomously and systemically for glucose tolerance because: 1) Their common site of detectable expression is the gonad sheath cells, yet they regulate membrane fluidity in intestinal cells and phospholipid composition in whole worms; and 2) Mosaic analysis shows that IGLR-2 can prevent glucose toxicity when expressed in only a subset of cells, namely the hypodermal cells that are descendants of the ABa and ABp blast cells and are an important site of fat storage and metabolic regulation [71–74]. The expression levels of PAQR-2 and IGLR-2 are likely tightly controlled: unpublished efforts to drive *paqr-2* expression from tissue-specific promoters have typically resulted in dead embryos, and it has also been quite difficult to generate transgenic animals carrying *iglr-2* expressing transgenic arrays, except when using small amounts of plasmids in the microinjection mix. A low level of expression in hypodermal cells could therefore have gone undetected in our previous efforts to describe the expression pattern of these two genes using reporter constructs. Note that PAQR-2 and IGLR-2 may also have important functions in the gonad sheath cells, where they are both predominantly expressed and where PAQR-2 depends on IGLR-2 for efficient expression. Previous studies have shown that the gonad sheath cells can regulate metabolism in other tissues, including phospholipid composition [31,75]. It is therefore plausible that PAQR-2 and IGLR-2 act in these cells and/or the hypodermis to monitor and regulate membrane homeostasis systemically.

### Considerations regarding the mammalian homologs

Mouse mutants lacking either or both AdipoR1 and AdipoR2 are without obvious phenotypes when maintained under normal conditions but develop metabolic syndrome symptoms when challenged with high fat diets [76]. This is analogous to the *C*. *elegans paqr-2* and *iglr-2* mutants that exhibit no severe phenotype under normal conditions but exhibit severe defects when challenged with exogenous glucose. The AdipoR1 and AdipoR2 genes are widely expressed in mammals and it is likely that they regulate metabolism in many cells of the body, though their functions are best described in metabolically active tissues such as liver, skeletal muscle and adipose tissue [7]. It will be interesting to determine whether they too regulate membrane fluidity in mammalian cells. The relevance of our findings for the pathologies seen in diabetic patients clearly remains to be investigated. That elevated glucose may impair membrane properties also in humans is however supported by the increased rigidity of membranes in erythrocytes and other cell types associated with high blood glucose [77–80], and reduced membrane fluidity is a proposed mechanism behind several of the cellular and vascular problems of diabetic patients [81]. It is also interesting to note that a decrease in membrane UFAs is a risk factor to develop overt diabetes [80].

In summary, we found that inclusion of glucose in the culture plates causes increased membrane rigidity in *C*. *elegans* lacking PAQR-2 or IGLR-2, and that an important function of these proteins is to counter such an effect by promoting fatty acid desaturation.

## Materials and Methods

### *C*. *elegans* strains and transgenes

The wild-type reference strain was the *C*. *elegans* Bristol variety strain, N2. Unless otherwise stated, strains were obtained from the *C*. *elegans* Genetics Center (CGC; MN, USA), and experiments were performed using the *E*. *coli* strain OP50 as food source, which was maintained by passaging either on NGM plates or liquid cultures in LB medium using standard protocols [82]; note that the effects of glucose reported here are even more pronounced when *C*. *elegans* is cultivated on fresh isolates of OP50 *E*. *coli* (obtained from the *C*.*elegans* Genetics Center) rather than multiply passaged OP50. The *paqr-2(tm3410)* and *iglr-2(et34)* mutant alleles were used in most experiments and are simply referred to as the *paqr-2* and *iglr-2* mutants. The *pPAQR-2*::*GFP* construct [13] and the *pfat-7*::*GFP (rtIs30)* carrying strain HA1842 (a kind gift from Amy Walker) [12] have been described elsewhere. *acdh-11(gk753061)* was a kind gift from Bob Horvitz [39].

### Glucose plates and assays

Glucose plates were prepared by adding glucose (1M, sterile filtered) into the cooled down NGM after autoclaving. For length measurement studies, synchronized L1s were plated onto glucose plates seeded with OP50. Worms were mounted and photographed 72 h later and the length of 20–25 worms was measured using ImageJ [83]. Scoring of fertile adults on glucose plates was performed 96 h after plating the L1s (n ≥ 100).

### Screen for novel mutants that are similar to *paqr-2(tm3410)*

Two strategies were used to isolate novel mutants that are phenotypically similar to *paqr-2(tm3410)* among the F2 progeny of EMS-mutagenized worms. In the first strategy, we isolated mutants with a tail tip defect then searched among those for mutants that also had the cold sensitivity phenotype. Approximately 11 000 mutagenized haploid genomes were screened in this way, yielding 9 promising mutants (alleles *et34*, *et35*, *et37-et43)*. In the second strategy, we isolated mutants with the cold sensitivity phenotype then searched among those for mutants that also had a tail tip defect. Approximately 70 000 mutagenized haploid genomes were screened in this way, yielding 1 mutant (allele *et36)*. Each mutant was outcrossed 4–6 times prior to whole-genome sequencing, and a minimum of 10 times prior to careful phenotypic characterization.

### Whole genome sequencing and mutant identification

The genomes of the novel mutants outcrossed 4 or 6 times were sequenced to a depth of 25–40x and differences between the reference N2 genome and that of the mutants were identified as previously described [18]. For each novel mutant, one or two mutation clusters, i.e. small genomic areas containing several mutations, were identified, which is in accordance to previous reports. These candidate mutations were tested experimentally as described in the text.

### Construction of plasmids

#### *iglr-2* cDNA

The RNA from N2 worms were isolated using Trizol (InVitrogen) and the cDNA was then synthesized using a Hig Capacity cDNA Reverse Transcription Kit (Applied Biosystems). The *iglr-2* cDNA (PCR product: 2416bp) was amplified using the primers 5'-gaacatcgcagatgtcttgat-3'and 5'-gatgatggcaccgattttggaa-3', which anneal just outside the predicted start and stop codons of the *iglr-2* gene. The amplified product was cloned in *pCR2*.*1-TOPO* (InVitrogen), and the positive clone was confirmed by sequencing.

#### *pIGLR*-*2*

The *iglr-2* operon rescue construct contains the *iglr-2* and *mrps-18A* genes along with 3 kb upstream and 1 kb downstream of the predicted operon. It was amplified from the *ZC262* fosmid using Platinum Taq DNA Polymerase High Fidelity (InVitrogen) and the amplified product (8 691 bp) was cloned in *pCR-XL-TOPO* (InVitrogen) to produce *pIGLR-2*. The following primers were used for the amplification: 5'-cagtagtcttcagaggccgaattg-3' and 5'-gacgtgacctacgtccctattttgc-3'.

#### *pIGLR*-*2*::*GFP*

*pIGLR-2*::*GFP* was constructed using Gibson assembly (NEB) with 4 fragments: *iglr-2* promoter (primers: 5’-ccagtgtgctggaattcgccctgacgtgacctacgtccctattttgca-3’ and 5’-aaaatacaaattttcgcatttcttcttttctttgtatcaagacatctgcgatgt-3’), *iglr-2* gene (5’- aaagaaaagaagaaatgcgaaaatttgtatttttcgtcgtagctattcttattca-3’ and 5’- cagtgaaaagttcttctcctttacttctcttttctggtggagaatct-3’), GFP (5’- agtaaaggagaagaacttttcactggagttgtcccaattcttgttg-3’ and 5’- ggttatagacaaacaaacaaaatgatttaaagaattatttgtatagttcatccatgcca-3’) and *iglr-2* 3’UTR together with the vector *pCR-XL-TOPO* (Invitrogen, 5’-aattctttaaatcattttgtttgtttgtctataaccattccaaaatcggtgcca-3’ and 5’-acgtcagggcgaattccagcacactggcggccgttactagtggat-3’). The GFP sequence was amplified using *pPD95*.*75* as template while all *iglr-2* fragments were amplified from *pIGLR-2*.

### Bimolecular fluorescence complementation (BiFC) constructs and analysis

As a template to make *pCE-IGLR2-VC155* a gene-cDNA hybrid retaining the first intron of *iglr-2* was constructed in *pUC19* using the following 5 fragments in a Gibson assembly: *iglr-2* promoter (primers: 5’- gacgttgtaaaacgacggccagtcctgcaggcaatgtccaaatccgaatccag-3’ and 5’- gctacgacgaaaaatacaaattttgcggccgctcgcatttattattgaattttttatg-3’), beginning of *iglr-2* gene until end of 2^nd^ exon (5’-cataaaaaattcaataataaatgcgagcggccgcaaaatttgtatttttcgtcgtagc-3’ and 5’-cagttccattcagagattccaaatcaccatctcggagaattatatgttctagttgcggaaatg-3’), *iglr-2* cDNA from exon 3 to the last coding amino acid (5’- catttccgcaactagaacatataattctccgagatggtgatttggaatctctgaatggaactg-3’ and 5’- ttacttgtcatcgtcatccttgtaatccttgtcatcgtcatccttgtaatccttgtcatcgtcatccttgtaatctctcttttctggtggagaatctgg-3’), *iglr-2* 3’UTR (5’-gagattacaaggatgacgatgacaaggattacaaggatgacgatgacaaggattacaaggatgacgatgacaagtaattctttaaatcattttgtttg-3’ and 5’-cacaggaaacagctatgaccatgattacgcccacgtggacaatgcttgaccgatcg-3’) and the *pUC19* vector (5’-gctatcacagttccgatcggtcaagcattgtccacgtgggcgtaatcatggtcatagctgtttc-3’ and 5’- ctggattcggatttggacattgcctgcaggactggccgtcgttttacaacgtc-3’). This plasmid was further used as template for amplification of the *iglr-2* gene-cDNA hybrid (5’- agattacgctcgaaaatttgtatttttcgtcgtagctattct-3’ and 5’- cgccacctccgctcccgccacctcctctcttttctggtggagaatct-3’) that was cloned into the *pCE-BiFC-VC155* vector[33] (5’-ggaggtggcgggagcggaggtggcgggagtgacaagcagaagaac-3’ and 5’- aattttcgagcgtaatctggaacatcgtatgggtacat-3’) using Gibson assembly (NEB).

As a template to make *pCE-VN173-PAQR2* a gene-cDNA hybrid retaining the first intron of *paqr-2* was constructed and assembled with *pUC19* using 5 fragment Gibson assembly: *paqr-2* promoter (primers: 5’- gacgttgtaaaacgacggccagtcctgcagggtctagatggaatggcttgaggatctcgc-3’ and 5’- cttcagcgtagtctgggacgtcgtatgggtacgcggccgcctccattttgttaaagctgaattttag-3’), beginning of *paqr-2* gene until end of 2^nd^ exon (5’- gcggccgcgtacccatacgacgtcccagactacgctgaagatgacgtggaatcggcaac-3’ and 5’- gataatattttctgcctctcggagcacatcagtaacactccgcaatgggctttgtagattactg-3’), *paqr-2* cDNA from exon 3 to the last coding amino acid (5’- cagtaatctacaaagcccattgcggagtgttactgatgtgctccgagaggcagaaaatattatc-3’ and 5’- tttaaaaataaaaaattggaaacaaatctacctcataaaccaacatccgccggtgtccagtc-3’), *paqr-2* 3’UTR (5’- gactggacaccggcggatgttggtttatgaggtagatttgtttccaattttttatttttaaa-3’ and 5’- cacaggaaacagctatgaccatgattacgccctgaggagcaacaagtgaacaatgtgagagaac-3’) and the *pUC19* vector (5’-gttctctcacattgttcacttgttgctcctcagggcgtaatcatggtcatagctgtttcctgtg-3’ and 5’-cctcaagccattccatctagaccctgcaggactggccgtcgttttacaacgtc-3’). This plasmid was further used as template for amplification of the *paqr-2* gene-cDNA hybrid (5’- ggtggcggaggttctggtggcggaggttctggtggcggaggttctgaggaagatgacgtggaatcggca-3’ and 5’- tcacttgtcatcgtcatccttgtaatccttgtcatcgtcatccttgtaatccttgtcatcgtcatccttgtaatctaaaccaacatccgccggtgt-3’) which was assembled with the VN173 fragment (5’- ggcccaccatggcatcaatggtgagcaagggcgagga-3’ and 5’- ctcagaacctccgccaccagaacctccgccaccagaacctccgccaccctcgatgttgtggcggat-3’) and the remaining *pCE-BiFC-VN173* vector [33] (5’- gattacaaggatgacgatgacaaggattacaaggatgacgatgacaaggattacaaggatgacgatgacaagtgagcggccgcaggatcca-3’ and 5’-cattgatgccatggtgggcccgcgggtacaattgctagcca-3’).

The *pCE-VN173-PAQR1* plasmid was constructed using Gibson assembly with 4 fragments: beginning of *paqr-1* gene including 1^st^ exon and intron (5’-ggtggcggaggttctggtggcggaggttctggtggcggaggttctaatccagatgaggtcaatcgag-3’ and 5’- gagtagaacacttcgattttgtcgccagtttttcttgcttccggatttttcacct-3’), *paqr-1* cDNA from exon 2 (5’- tcacttgtcatcgtcatccttgtaatccttgtcatcgtcatccttgtaatccttgtcatcgtcatccttgtaatctctaactggacattgttcgttcaga-3’ and 5’-aaaaactggcgacaaaatcgaagtgttctactcccgcaaaacaacggtcgt-3’), VN173 (5’- ggcccaccatggcatcaatggtgagcaagggcgagga-3’ and 5’- ctcagaacctccgccaccagaacctccgccaccagaacctccgccaccctcgatgttgtggcggat-3’) and the remaining *pCE-BiFC-VN173* vector[33] (5’- gattacaaggatgacgatgacaaggattacaaggatgacgatgacaaggattacaaggatgacgatgacaagtgagcggccgcaggatcca-3’ and 5’-cattgatgccatggtgggcccgcgggtacaattgctagcca-3’).

The different combinations of BiFC plasmids were injected into N2 worms at 15 ng/μl each, together with *pRF4(rol-6)* at 100 ng/μl as previously described [34]. Expression of the BiFC constructs were induced by heat shocks of 2.5 h and 1.5 h at 33°C, with 2 h recovery at 20°C in between. Scoring of fluorescence was preformed after 16 h of recovery at 20°C.

The *paqr-2(et36)* constructs were made by modification of *pPAQR-2*::*GFP* and *pCE-VN173-PAQR2* using the Q5 Site-Directed Mutagenesis Kit (NEB) with primers 5’-caaaataacgaatacctccgt-3’ and 5’-aagccattcgggtagagt-3’.

### Lipidomics

Samples were composed of synchronized L4 larvae (one 9 cm diameter plate/sample) grown on NGM or NGM containing 20 mM glucose overnight. Worms were washed 3 times with M9, pelleted and stored at -80°C until analysis. For lipid extraction, the pellet was sonicated for 10 minutes in methanol and then extracted according to published methods [84]. Internal standards were added in the chloroform phase during the extraction. Lipid extracts were evaporated and reconstituted in chloroform:methanol [1:2] with 5 mM ammonium acetate. This solution was infused directly (shotgun approach) into a QTRAP 5500 mass spectrophotometer (ABSciex, Toronto, Canada) equipped with a Nanomate Triversa (Advion Bioscience, Ithaca, NY) as described previously [85]. Phospholipids were measured using multiple precursor ion scanning [86,87]. The data was evaluated using the LipidProfiler software [86].

### Fluorescence Recovery after Photobleaching (FRAP) analysis

FRAP experiments were carried out using a Zeiss LSM700inv laser scanning confocal microscope with a Plan-Apochromat 20X objective lens. The membranes of intestinal cells expressing the *pGLO-1P*::*GFP-CAAX* reporter [88] were photobleached over a circular region (7 pixels radius) using 10 iterations of the 488 nm laser with 100% laser power transmission. Images were collected at a 12-bit intensity resolution over 512×512 pixels (digital zoom 6X) using a pixel dwell time of ∼1 μsec, and were all acquired under identical settings. The fluorescence recovery of the bleached region was calculated as follows. Firstly, all fluorescence values were adjusted to compensate for the slight and gradual bleaching caused by repetitive scanning and imaging. This was done by adjusting fluorescence values by the slope of the decreasing fluorescence in a reference non-photobleached region. In a next step, the lowest intensity value (immediately after bleaching) was identified and this value was subtracted from all intensities, thus setting the post-bleach fluorescence as zero. The average intensities of the five measurements that precede the bleaching were then determined, establishing a pre-bleach value; all intensities were normalized by dividing by that value. The average of the last five measurements (assumed to approximate the plateau of recovery) represent the maximum recovery and corresponds to the mobile fraction. The halftime of recovery is the time point where the fluorescence recovered to half of the maximum recovery. The data are expressed as means ± S.E. Experiments related to temperature (15–20°C) were performed on L4 larvae grown overnight at two different temperatures. For the glucose experiments, L1s grown overnight with or without 20 mM glucose were used. For each strain, N>5 worms were immobilized using 100 mM levamisole prior to analysis.

### Mosaic analysis

The plasmid *pTG96* carrying the SUR-5GFP(NLS) [51], a kind gift from Prof. Han, Boulder, Colorado, was co-injected into the gonad syncytium of wild-type worms at a concentration of 50 ng/μl together with 5 ng/μl of *pIGLR-2* to establish a transgenic line. The extrachromosomal array was then crossed into the *iglr-2* mutant background, and these transgenic worms were bleached and their eggs allowed to hatch overnight in M9 to produce synchronized L1s that were transferred to NGM plates containing 20 mM glucose. Worms that grew to into adults were scored 72 hours later.

### Other methods

Generation of transgenic animals, self brood size assay, growth rate assay, 15°C growth assay, scoring of *paqr-2* tail tip phenotype, RNAi feeding, and quantification of *pfat-7*::*GFP* expression were performed as previously described [18].

### Statistics

Error bars for worm length measurements show the standard error of the mean, and *t-tests* were used to identify significant differences between worm lengths. Error bars for the percentage of fertile adults and the frequency of the tail tip defect show the 95% confidence interval determined using *Z-tests*.

## Supporting Information

S1 Fig Sensitivity of *paqr-2* and *iglr-2* to various sugars and characterization of other *paqr-*2 and *iglr-*2 mutant alleles. **(A)** The *paqr-2* and *iglr-2* single and double mutants are similarly sensitive to several types of monosaccharides, but most sensitive to glucose. Apparent differences between the mutant genotypes reflect variation among treated worms, probably due to exposing the worms to concentrations near the toxic dose threshold for a given sugar, as is the case for sorbitol and galactose. We measured the lengths of 20 worms per condition and a few outliers can indeed give a misleading impression. In other repeats of this experiment, it was sometimes *iglr-2* or *paqr-2*, rather than the double mutant, that appeared more sensitive. **(B)** Fraction of worms that grow into fertile adults after being incubated as L1s on 20 mM glucose then transferred to normal plates after 3, 6, 12, 24 and 48 hours and allowed to grow a further 72 hours. Note that the toxic effect of 20 mM glucose on the mutants is reversible within 3 hours, but less so after longer exposures. **(C-F)** The *paqr-2* alleles *et35* and *et36*, as well as the *iglr-2* alleles *et37* and *et38*, are sensitive to 15°C cultivation **(C),** exhibit excess of saturated fatty acids in their phosphatitylcholines (PC) and phosphatidylethanolamines (PE) **(D),** show a decrease in expression of *pfat-7*::*GFP* **(E),** and are sensitive to glucose **(F)**, and. Note that *et36* is a weaker allele in terms of glucose and 15°C sensitivity. **(G-H)** The *paqr-2* and *iglr-2* single and double mutants also have similar brood sizes **(G)** and growth rates **(H)** when cultivated on normal plates.
(TIF)

S2 Fig Experimental confirmation of *iglr-2*. *iglr-2* mutants carrying a wild-type *iglr-2* transgene are able to grow at 15°C **(A** and **C)** and have a normal tail tip morphology **(B** and **C)**. **(D-F)** RNAi against *iglr-2* causes wild-type worms to exhibit a 15°C growth defect and a withered tail tip phenotype indistinguishable from that of *iglr-2* mutant worms.
(TIF)

S3 Fig PAQR-2 depends on IGLR-2 for localization to gonad sheath cell membranes. **(A)** Transgenic worms of the indicated genotypes carrying either *pIGLR-2*::*GFP* or *pPAQR-2*::*GFP* were photographed using DIC optics (left panels) or using epifluorescence to visualize the GFP-tagged translational reporter (right panels). In the wild-type genetic background (N2), both reporters are expressed on membranes of the somatic gonad sheath cells (the frequency of transgenic worms with GFP-positive somatic gonads are indicated). Expression of the *iglr-2* reporter is also frequent and strong in the *paqr-2* mutant background. In contrast, expression of the *paqr-2* reporter is dramatically reduced both in terms of frequency and intensity in the *iglr-2* mutant background. **(B)** The *paqr-2(et36)* allele encodes a PAQR-2 protein that still localizes to the gonad sheath cell (upper panels) and interacts with IGLR-2 when co-expressed in intestinal cells as determined using BiFC (lower panels). Arrowheads outline the gonad sheath cells while asterisks indicate BiFC signal in intestinal membranes. Scale bars: 20 μm.
(TIF)

S4 Fig Effect of *paqr-2* suppressors on glucose and cold sensitivity. **(A)** The *et7* and *et13 gof* alleles of *nhr-49* are potent suppressors, the *et11 lof* allele of *cept-1* is a partial suppressor, and the *lof* allele of *sams-1* is not a suppressor of glucose sensitivity in the *paqr-2* mutant. The *acdh-11(gk753061)* mutation does not suppresses the glucose sensitivity of the *paqr-2* mutant **(B)**, but suppresses the 15°C growth and tail tip defects **(C)**. **(D)** The *nhr-49(et8)*, *cept-1(et10)* and *hacd-1(et12)* mutations are effective suppressors of the 15°C growth defect of the *paqr-2* and *iglr-2* single and double mutants. **(E)** Nomarski images of wild-type and *lof* mutants grown with or without glucose. Note that the *mdt-15* mutant grows to adulthood while on glucose but produces only dead eggs (indicated by arrowheads).
(TIF)

S5 Fig The *paqr-2* and *iglr-2* mutants are unable to maintain membrane fluidity at low temperatures. **(A-C)** FRAP analysis shows that wild-type worms cultivated overnight at 15°C have the same membrane fluidity as worms grown at 20°C but that *paqr-2* and *iglr-2* worms cultivated overnight at 15°C have a marked decrease in membrane fluidity. T_half_ values for 15°C are provided and expressed in seconds needed to reach half off the maximal fluorescence recovery. **(D)** Overnight incubation of larvae in the presence of 20 mM glucose causes a much stronger increase in SFAs among the PEs of *paqr-2* and *iglr-2* single and double mutants than in wild-type N2 worms. * *p* ≤ 0.05 and *** p *≤* 0.001.
(TIF)

S1 Table PC composition in wild type and mutants. Each row indicates the fraction (mol%) of fatty acids in PC that had the indicated number of carbon atoms and double bonds.
(PDF)

S2 Table PE composition in wild type and mutants. Each row indicates the fraction (mol%) of fatty acids in PE that had the indicated number of carbon atoms and double bonds.
(PDF)
