# Correction: *Caenorhabditis elegans* PAQR-2 and IGLR-2 Protect against Glucose Toxicity by Modulating Membrane Lipid Composition

**DOI:** 10.1371/journal.pgen.1006112

**Published:** 2016-06-03

**Authors:** Emma Svensk, Ranjan Devkota, Marcus Ståhlman, Parmida Ranji, Manish Rauthan, Fredrik Magnusson, Sofia Hammarsten, Maja Johansson, Jan Borén, Marc Pilon

Some ORF names, lengths and mutations in Table 1 are incorrect. Specifically, the ORF names and ORF lengths listed for the *paqr-2(et36)* and *paqr-2(et35)* alleles, and the ORF names and mutations listed for the *iglr-2(et34)*, *iglr-2(et37)* and *iglr-2(et38)* alleles. The shift by one amino acid position in the mutation column also applies to the *iglr-2* mutations indicated in Fig 2. Please see the corrected Table 1 and Fig 2 here.

**Table 1:** Description of the novel *paqr-2* and *iglr-2* alleles.

*Gene(allele)*	ORF name	ORF length	Mutation
*paqr-2(et36)*	Y32H12A.5	581 aa	*D(GAT)282N(AAT)*
*paqr-2(et35)*	Y32H12A.5	581 aa	*G(GGA)533R(AGA)*
*iglr-2(et34)*	ZC262.3a	773 aa	*W(TGG)84STOP(TAG)*
*iglr-2(et37)*	ZC262.3a	773 aa	*G(GGT)498D(GAT)*
*iglr-2(et38)*	ZC262.3a	773 aa	*Q(CAA)594STOP(TAA)*

**Fig 2:**
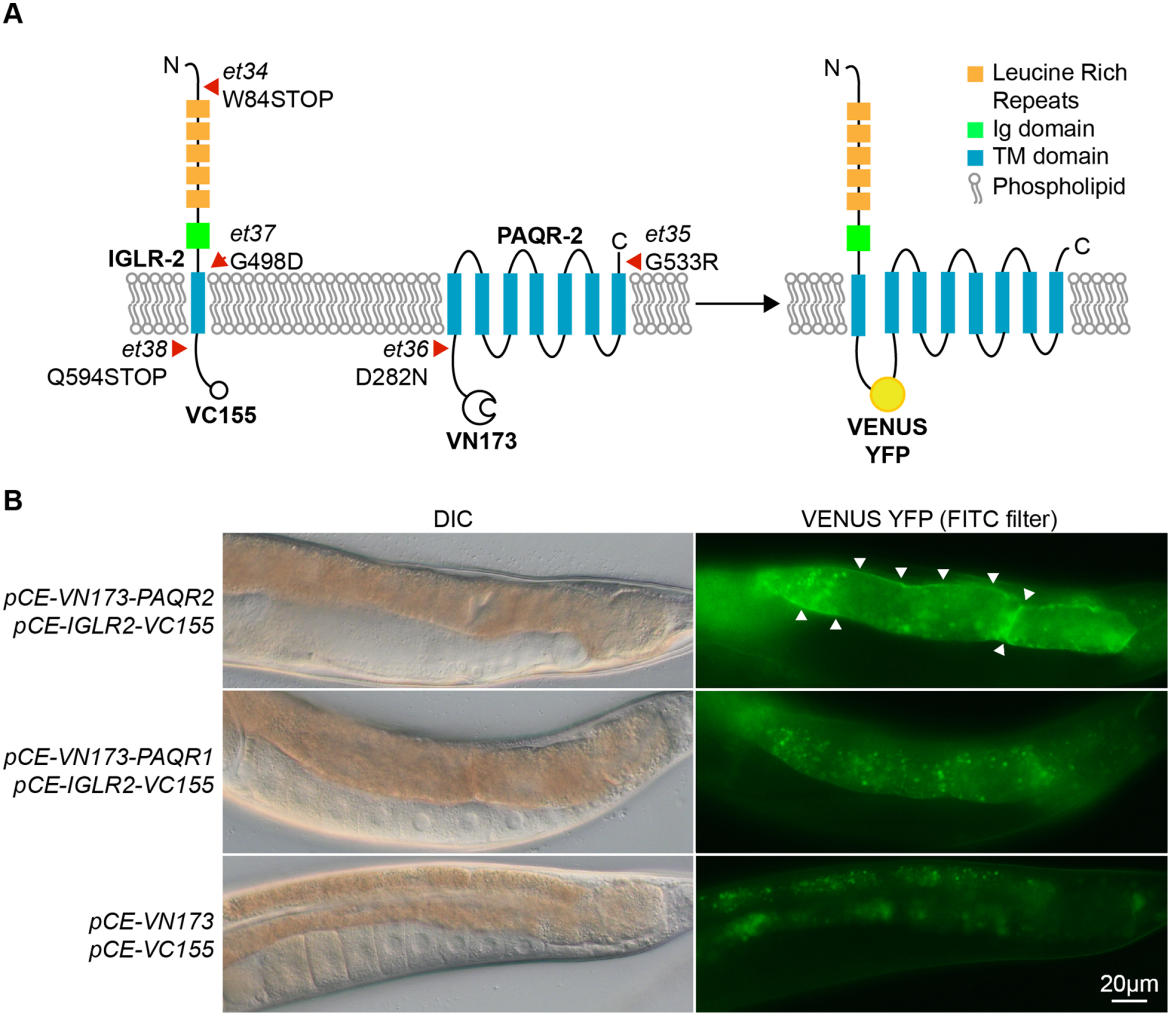
Novel alleles of PAQR-2 and IGLR-2, and interaction of IGLR-2 with PAQR-2. **(A)** Schematic structures of the IGLR-2 and PAQR-2 proteins, with novel mutations indicated by red arrowheads. The VC155 and VN173 fragments added to the C and N terminal ends of IGLR-2 and PAQR-2, respectively, allows reconstitution of a full and fluorescent VENUS YFP protein if the two proteins come into close proximity. **(B)** Result of the BiFC experiment showing that IGLR-2 and PAQR-2 contact each other on cellular membranes. The top two panels show a transgenic worm co-expressing the fusion proteins depicted in **(A)**; note the clear membrane-localized fluorescence indicative of IGLR-2 and PAQR-2 interaction. The middle two panels show a transgenic worm co-expressing the tagged IGLR-2 and a tagged PAQR-1 protein; note that only autofluorescent gut granules emit a signal, indicating that IGLR-2 and PAQR-1 do not interact with each other. The bottom two panels show a transgenic animal carrying the two empty vectors used in the BiFC experiments; note again that only autofluorescent gut granules emit a signal.
