# Supplementary figures and images for: Caenorhabditis elegans PAQR-2 and IGLR-2 Protect against Glucose Toxicity by Modulating Membrane Lipid Composition
Source: PLoS Genet. 2016 Apr 15;12(4):e1005982. doi: 10.1371/journal.pgen.1005982 (PMC4833288; doi:10.1371/journal.pgen.1005982)

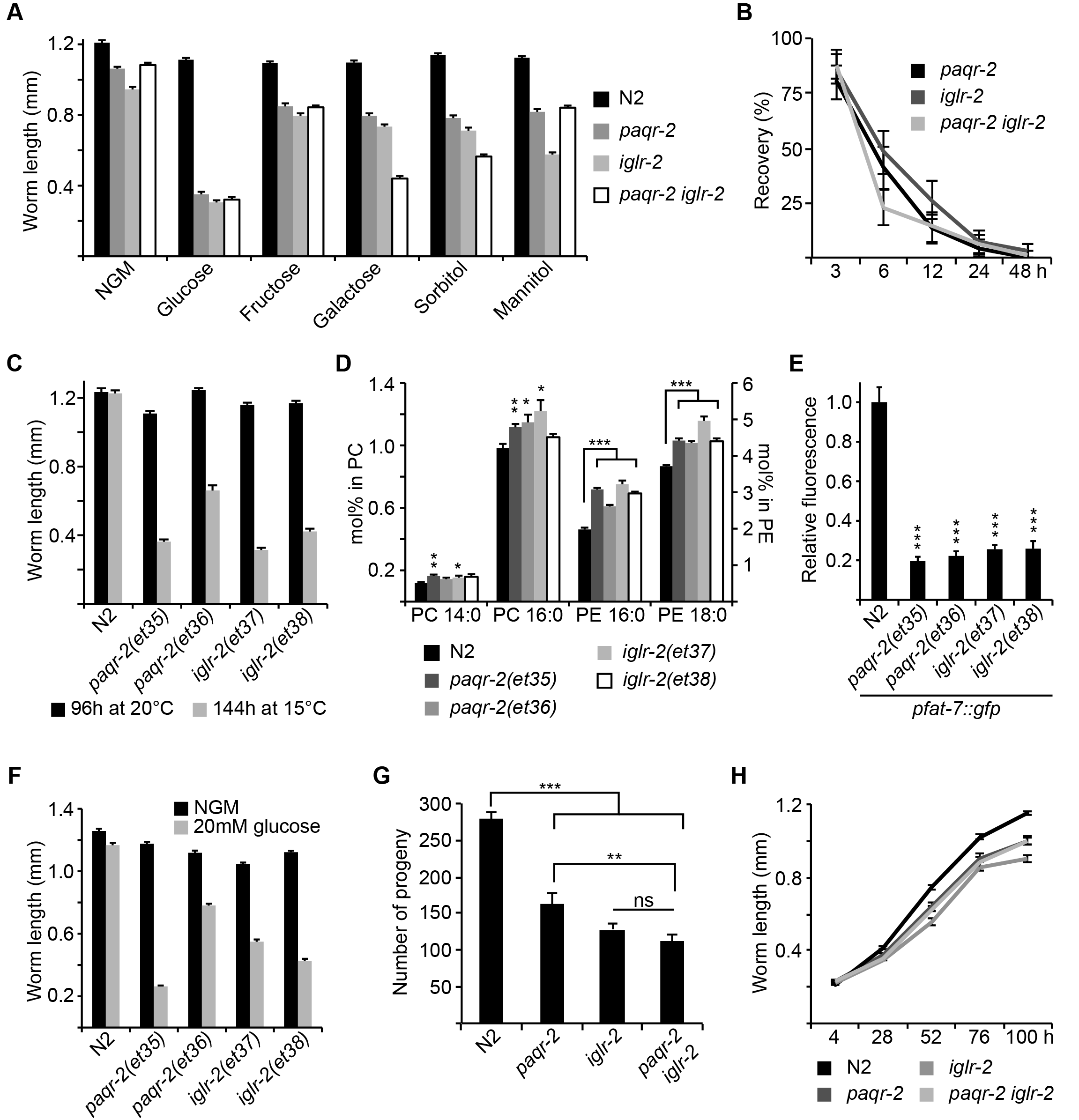

Supplement: S1 Fig — (A) The paqr-2 and iglr-2 single and double mutants are similarly sensitive to several types of monosaccharides, but most sensitive to glucose. Apparent differences between the mutant genotypes reflect variation among treated worms, probably due to exposing the worms to concentrations near the toxic dose threshold for a given sugar, as is the case for sorbitol and galactose. We measured the lengths of 20 worms per condition and a few outliers can indeed give a misleading impression. In other repeats of this experiment, it was sometimes iglr-2 or paqr-2, rather than the double mutant, that appeared more sensitive. (B) Fraction of worms that grow into fertile adults after being incubated as L1s on 20 mM glucose then transferred to normal plates after 3, 6, 12, 24 and 48 hours and allowed to grow a further 72 hours. Note that the toxic effect of 20 mM glucose on the mutants is reversible within 3 hours, but less so after longer exposures. (C-F) The paqr-2 alleles et35 and et36, as well as the iglr-2 alleles et37 and et38, are sensitive to 15°C cultivation (C), exhibit excess of saturated fatty acids in their phosphatitylcholines (PC) and phosphatidylethanolamines (PE) (D), show a decrease in expression of pfat-7::GFP (E), and are sensitive to glucose (F), and. Note that et36 is a weaker allele in terms of glucose and 15°C sensitivity. (G-H) The paqr-2 and iglr-2 single and double mutants also have similar brood sizes (G) and growth rates (H) when cultivated on normal plates. (TIF) [file pgen.1005982.s001.tif]

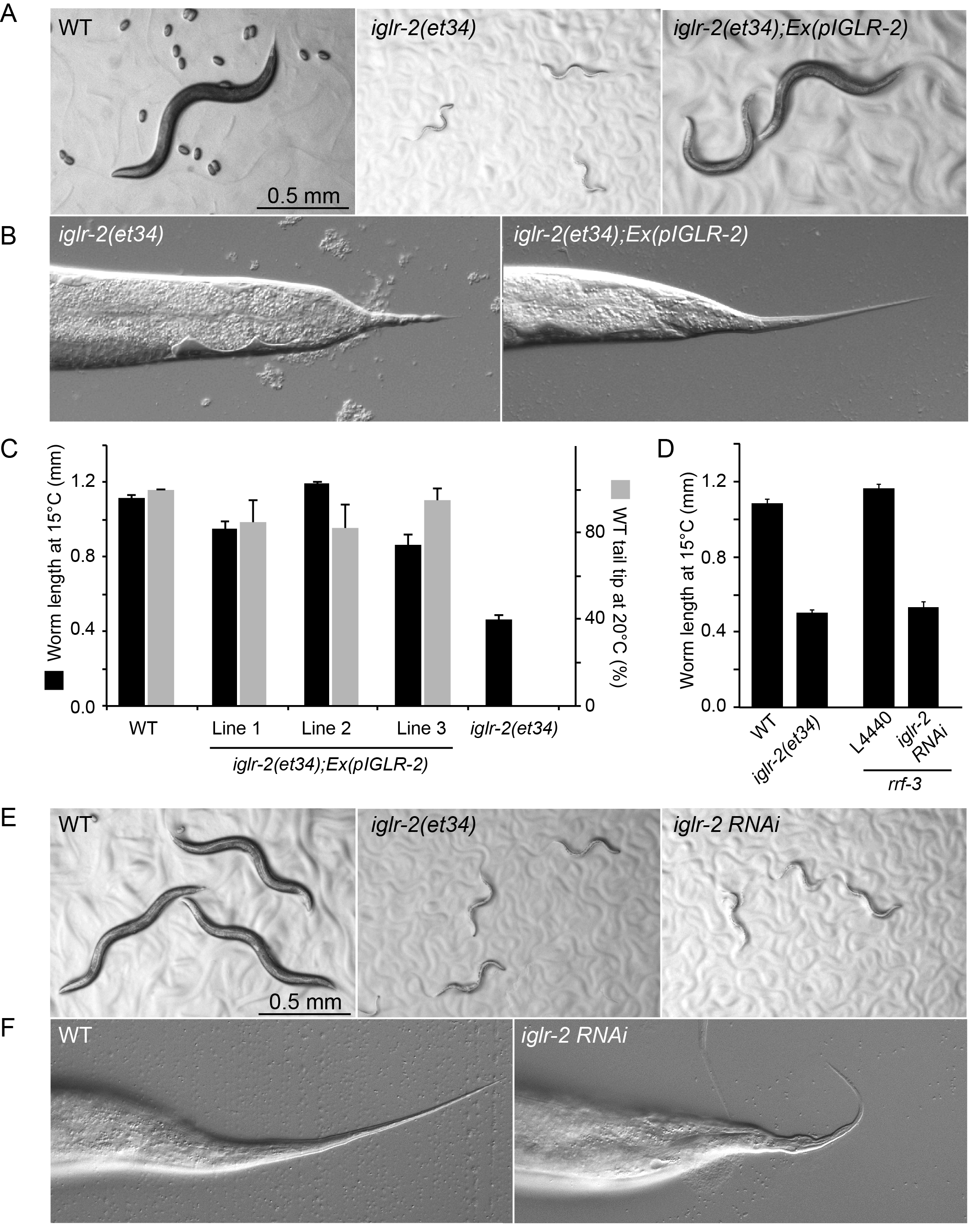

Supplement: S2 Fig — iglr-2 mutants carrying a wild-type iglr-2 transgene are able to grow at 15°C (A and C) and have a normal tail tip morphology (B and C). (D-F) RNAi against iglr-2 causes wild-type worms to exhibit a 15°C growth defect and a withered tail tip phenotype indistinguishable from that of iglr-2 mutant worms. (TIF) [file pgen.1005982.s002.tif]

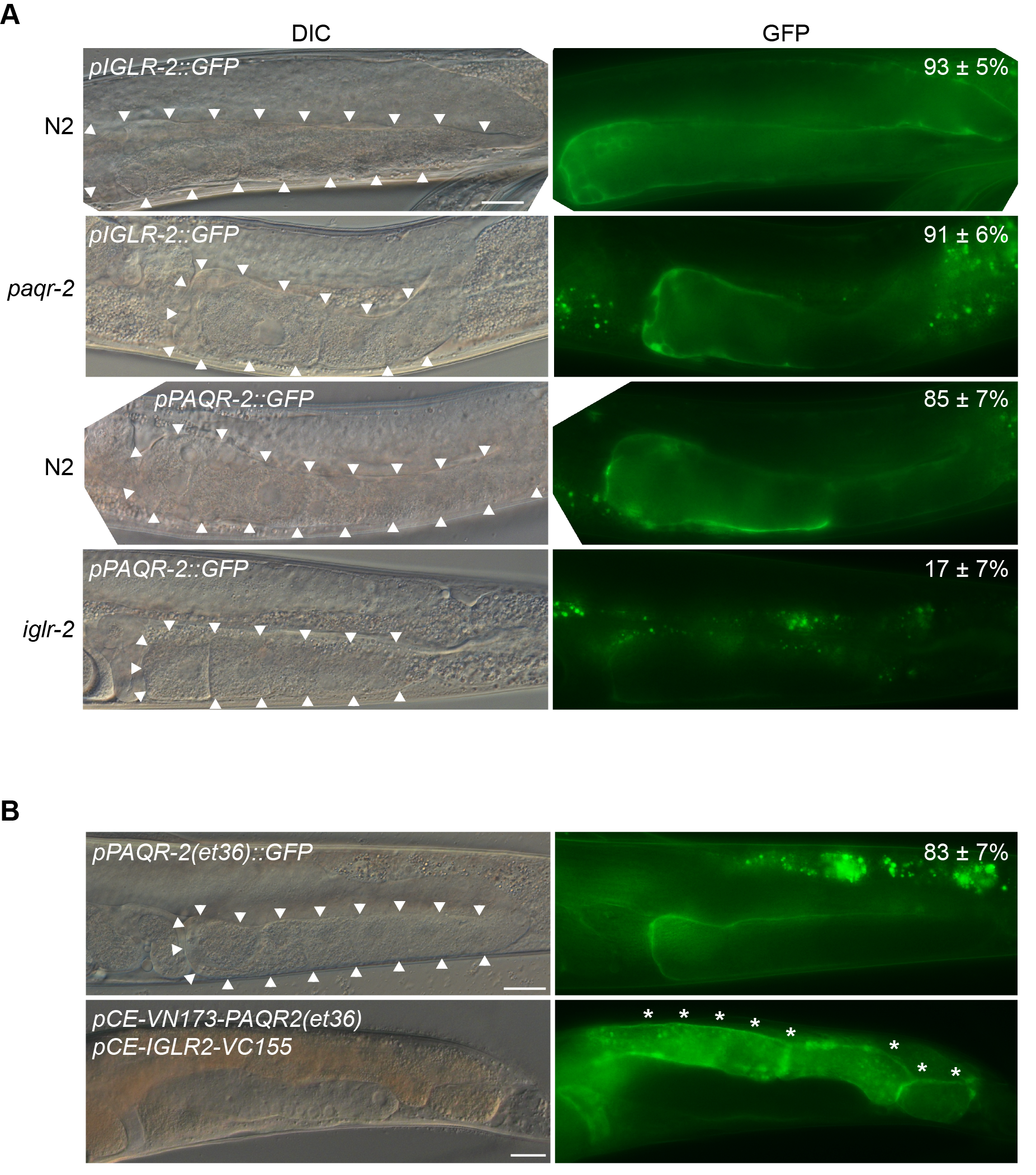

Supplement: S3 Fig — (A) Transgenic worms of the indicated genotypes carrying either pIGLR-2::GFP or pPAQR-2::GFP were photographed using DIC optics (left panels) or using epifluorescence to visualize the GFP-tagged translational reporter (right panels). In the wild-type genetic background (N2), both reporters are expressed on membranes of the somatic gonad sheath cells (the frequency of transgenic worms with GFP-positive somatic gonads are indicated). Expression of the iglr-2 reporter is also frequent and strong in the paqr-2 mutant background. In contrast, expression of the paqr-2 reporter is dramatically reduced both in terms of frequency and intensity in the iglr-2 mutant background. (B) The paqr-2(et36) allele encodes a PAQR-2 protein that still localizes to the gonad sheath cell (upper panels) and interacts with IGLR-2 when co-expressed in intestinal cells as determined using BiFC (lower panels). Arrowheads outline the gonad sheath cells while asterisks indicate BiFC signal in intestinal membranes. Scale bars: 20 μm. (TIF) [file pgen.1005982.s003.tif]

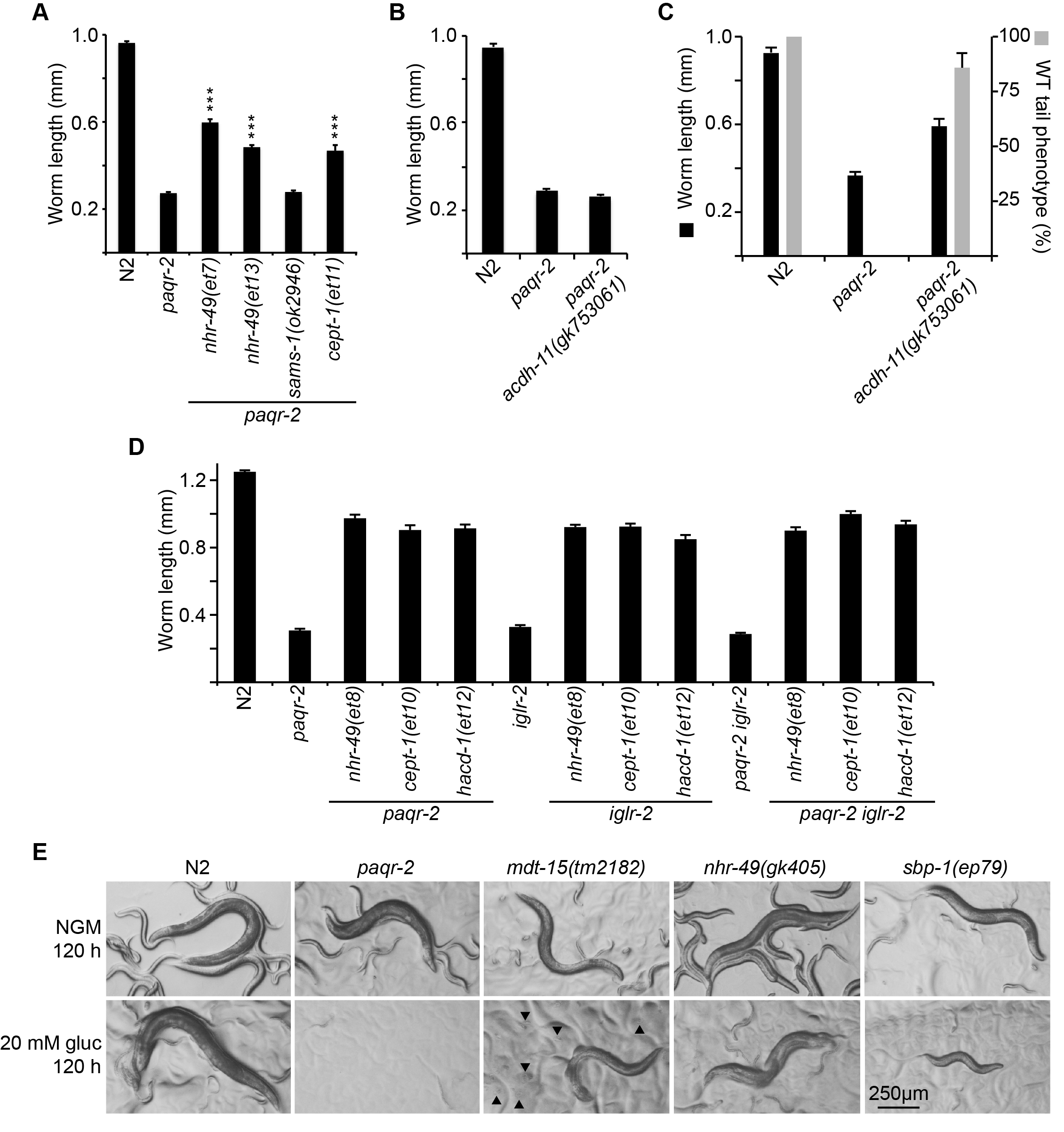

Supplement: S4 Fig — (A) The et7 and et13 gof alleles of nhr-49 are potent suppressors, the et11 lof allele of cept-1 is a partial suppressor, and the lof allele of sams-1 is not a suppressor of glucose sensitivity in the paqr-2 mutant. The acdh-11(gk753061) mutation does not suppresses the glucose sensitivity of the paqr-2 mutant (B), but suppresses the 15°C growth and tail tip defects (C). (D) The nhr-49(et8), cept-1(et10) and hacd-1(et12) mutations are effective suppressors of the 15°C growth defect of the paqr-2 and iglr-2 single and double mutants. (E) Nomarski images of wild-type and lof mutants grown with or without glucose. Note that the mdt-15 mutant grows to adulthood while on glucose but produces only dead eggs (indicated by arrowheads). (TIF) [file pgen.1005982.s004.tif]

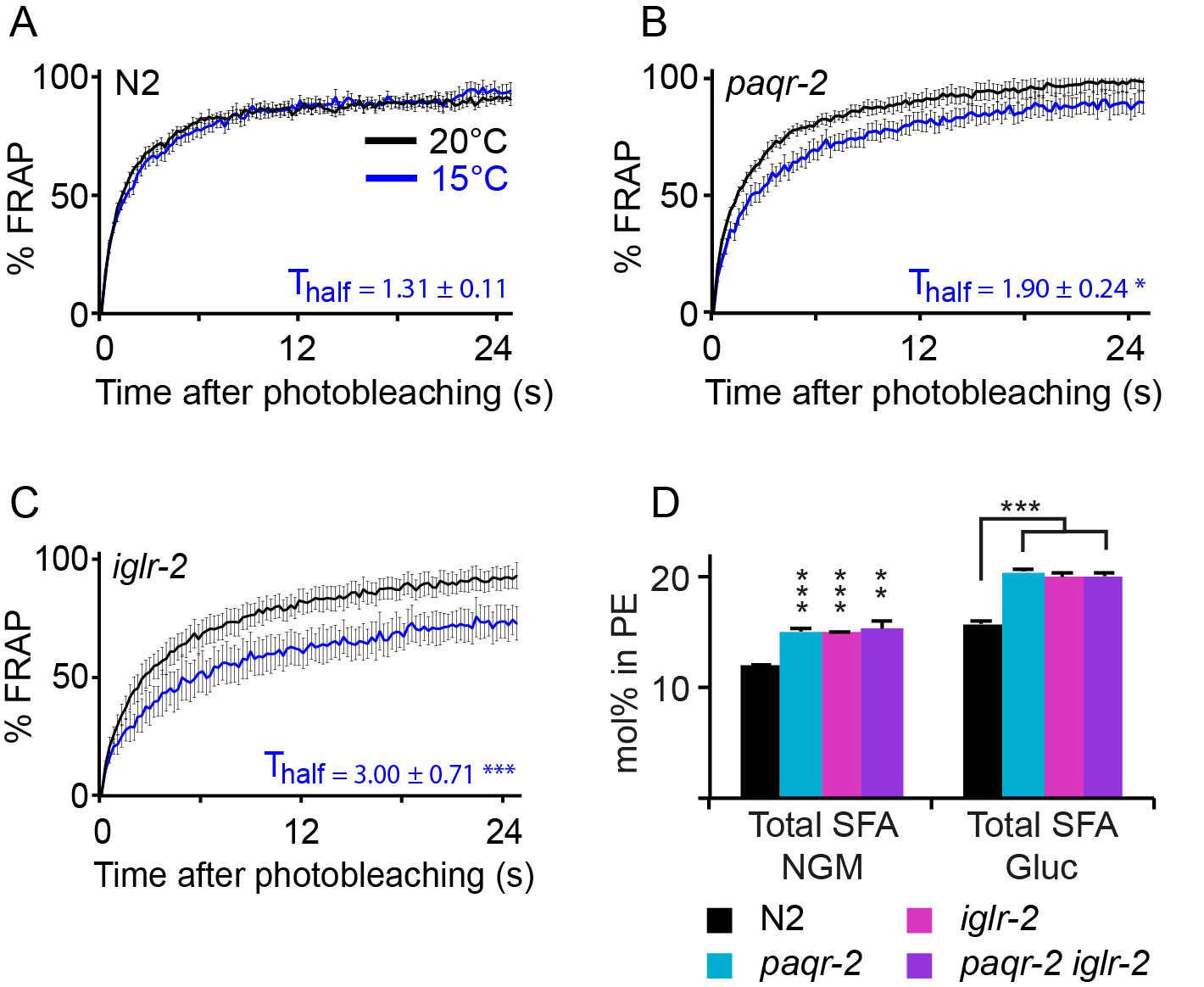

Supplement: S5 Fig — (A-C) FRAP analysis shows that wild-type worms cultivated overnight at 15°C have the same membrane fluidity as worms grown at 20°C but that paqr-2 and iglr-2 worms cultivated overnight at 15°C have a marked decrease in membrane fluidity. Thalf values for 15°C are provided and expressed in seconds needed to reach half off the maximal fluorescence recovery. (D) Overnight incubation of larvae in the presence of 20 mM glucose causes a much stronger increase in SFAs among the PEs of paqr-2 and iglr-2 single and double mutants than in wild-type N2 worms. * p ≤ 0.05 and *** p ≤ 0.001. (TIF) [file pgen.1005982.s005.tif]
