# Supplementary material for: Caenorhabditis elegans PAQR-2 and IGLR-2 Protect against Glucose Toxicity by Modulating Membrane Lipid Composition
Source: PLoS Genet. 2016 Apr 15;12(4):e1005982. doi: 10.1371/journal.pgen.1005982 (PMC4833288; doi:10.1371/journal.pgen.1005982)
Supplement: S1 Table — Each row indicates the fraction (mol%) of fatty acids in PC that had the indicated number of carbon atoms and double bonds. (PDF) [file pgen.1005982.s006.pdf]

Table S1: FA composition in PC (mol%)

	N2	*paqr-2(tm3410)*	*paqr-2(et35)*	*paqr-2(et36)*	*iglr-2(et34)*	*iglr-2(et37)*	*iglr-2(et38)*	*paqr-2(tm3410) iglr-2(et34)*
PC FA 14:0	0.12±0.01	0.18±0.01***	0.16±0.01**	0.14±0.01	0.16±0.01**	0.15±0.01*	0.16±0.02	0.18±0.01***
PC FA 15:0	1.72±0.05	1.82±0.04	1.82±0.02	1.79±0.02	1.70±0.04	1.93±0.03**	1.80±0.06	1.69±0.07
PC FA 16:0	0.98±0.03	1.31±0.05***	1.12±0.02**	1.15±0.05*	1.25±0.07**	1.22±0.07*	1.05±0.02	1.17±0.03**
PC FA 17:0	2.74±0.07	2.29±0.05***	2.66±0.05	2.74±0.03	2.44±0.08*	2.77±0.04	2.73±0.10	2.10±0.03***
PC FA 18:0	1.54±0.08	1.87±0.12*	1.59±0.02	1.57±0.10	1.60±0.10	1.87±0.11*	1.62±0.06	1.72±0.16
PC FA 19:0	0.09±0.01	0.07±0.00	0.07±0.00*	0.09±0.01	0.08±0.01	0.09±0.00	0.09±0.01	0.07±0.01
PC FA 16:1	2.19±0.05	2.55±0.02***	2.57±0.05***	2.15±0.03	2.92±0.10***	2.32±0.08	2.22±0.08	2.96±0.09***
PC FA 17:1	6.81±0.05	6.68±0.10	6.19±0.18**	6.97±0.15	6.69±0.08	6.60±0.10	6.79±0.12	6.68±0.18
PC FA 18:1	30.29±0.29	30.48±0.25	30.80±0.46	31.24±0.19*	32.36±0.15***	30.71±0.25	29.97±0.61	30.93±0.54
PC FA 19:1	2.67±0.04	1.64±0.03***	2.12±0.08***	2.49±0.03**	1.90±0.05***	2.17±0.05***	2.64±0.07	1.57±0.05***
PC FA 20:1	0.34±0.01	0.25±0.00***	0.29±0.02*	0.31±0.00**	0.26±0.01***	0.28±0.00***	0.33±0.01	0.25±0.01***
PC FA 16:2	0.33±0.01	0.37±0.01**	0.39±0.01**	0.36±0.01*	0.48±0.02***	0.38±0.01*	0.37±0.01*	0.43±0.02**
PC FA 18:2	7.47±0.27	5.78±0.16***	6.07±0.03***	6.08±0.13**	5.25±0.15***	5.28±0.22***	5.10±0.06***	6.66±0.17*
PC FA 18:3	3.72±0.07	4.60±0.03***	4.36±0.04***	3.78±0.06	4.78±0.05***	4.22±0.07***	4.15±0.07**	4.94±0.08***
PC FA 20:2	0.32±0.01	0.30±0.01	0.33±0.02	0.30±0.02	0.26±0.01**	0.27±0.02	0.32±0.01	0.31±0.02
PC FA 20:3	4.13±0.04	4.67±0.09***	4.47±0.05***	4.07±0.07	4.27±0.04*	4.49±0.08**	4.51±0.15*	4.64±0.15*
PC FA 20:4	8.46±0.08	8.79±0.16	8.94±0.17*	8.43±0.21	8.40±0.10	9.21±0.08***	9.01±0.30	8.83±0.26
PC FA 20:5	26.08±0.43	26.35±0.26	26.04±0.23	26.35±0.08	25.18±0.27	26.04±0.31	27.13±0.52	24.85±0.35

*p<0.05, **p<0.01, ***p<0.001
