# Supplementary material for: Caenorhabditis elegans PAQR-2 and IGLR-2 Protect against Glucose Toxicity by Modulating Membrane Lipid Composition
Source: PLoS Genet. 2016 Apr 15;12(4):e1005982. doi: 10.1371/journal.pgen.1005982 (PMC4833288; doi:10.1371/journal.pgen.1005982)
Supplement: S2 Table — Each row indicates the fraction (mol%) of fatty acids in PE that had the indicated number of carbon atoms and double bonds. (PDF) [file pgen.1005982.s007.pdf]

Table S2: FA composition in PE (mol%)

	N2	*paqr-2(tm3410)*	*paqr-2(et35)*	*paqr-2(et36)*	*iglr-2(et34)*	*iglr-2(et37)*	*iglr-2(et38)*	*paqr-2(tm3410) iglr-2(et34)*
PE FA 14:0	0.02±0.00	0.03±0.00*	0.02±0.00	0.02±0.00	0.03±0.00*	0.02±0.00	0.02±0.00	0.02±0.00
PE FA 15:0	0.90±0.02	1.36±0.01***	1.17±0.04***	1.11±0.03***	1.14±0.02***	1.18±0.01***	1.17±0.01***	1.21±0.03***
PE FA 16:0	1.98±0.05	3.58±0.05***	3.08±0.04***	2.61±0.04***	3.53±0.11***	3.22±0.10***	2.97±0.05***	3.75±0.05***
PE FA 17:0	4.52±0.08	5.82±0.10***	5.83±0.08***	5.60±0.08***	5.76±0.05***	5.76±0.08***	5.93±0.08***	5.38±0.07***
PE FA 18:0	3.71±0.03	4.65±0.12***	4.42±0.06***	4.35±0.05***	4.62±0.17***	4.96±0.12***	4.40±0.08***	4.56±0.05***
PE FA 19:0	0.33±0.01	0.29±0.01	0.29±0.00	0.34±0.00	0.32±0.01	0.32±0.01	0.31±0.01	0.30±0.01
PE FA 20:0	0.29±0.01	0.27±0.00**	0.32±0.01*	0.32±0.01*	0.30±0.01	0.31±0.01*	0.31±0.00	0.31±0.01
PE FA 16:1	1.92±0.06	2.04±0.02	1.94±0.02	1.79±0.03	2.20±0.10*	1.80±0.03	1.87±0.04	2.30±0.03***
PE FA 17:1	8.24±0.09	7.15±0.09***	6.39±0.23***	7.66±0.10**	7.34±0.13***	6.78±0.09***	7.30±0.24**	7.38±0.22**
PE FA 18:1	52.13±0.35	51.42±0.21	54.07±0.20**	54.39±0.27***	53.50±0.36*	54.04±0.26**	53.94±0.26**	52.46±0.17
PE FA 19:1	2.17±0.06	1.78±0.03***	1.84±0.06**	2.08±0.03	1.85±0.04**	1.90±0.04**	2.07±0.04	1.66±0.04***
PE FA 20:1	0.78±0.02	0.78±0.01	0.89±0.01**	0.89±0.01***	0.82±0.01	0.84±0.01*	0.88±0.02**	0.70±0.01**
PE FA 16:2	0.16±0.01	0.17±0.02	0.17±0.01	0.16±0.01	0.20±0.01*	0.16±0.01	0.16±0.00	0.18±0.01
PE FA 18:2	8.36±0.19	7.77±0.19	7.19±0.07***	6.30±0.13***	6.63±0.16***	6.23±0.20***	6.29±0.07***	7.99±0.21
PE FA 18:3	1.79±0.03	1.95±0.03*	1.71±0.02	1.56±0.02***	1.86±0.01	1.61±0.03**	1.65±0.02**	1.92±0.03*
PE FA 20:2	0.65±0.02	0.61±0.02	0.68±0.00	0.55±0.01**	0.55±0.03*	0.55±0.03*	0.64±0.02	0.63±0.01
PE FA 20:3	2.71±0.03	2.88±0.08	2.53±0.02**	2.30±0.02***	2.40±0.05**	2.52±0.04*	2.54±0.01**	2.43±0.06**
PE FA 20:4	3.51±0.07	3.05±0.09**	2.84±0.03***	2.88±0.04***	2.66±0.04***	2.99±0.08**	2.94±0.02***	2.67±0.03***
PE FA 20:5	5.82±0.11	4.42±0.14***	4.61±0.05***	5.08±0.09***	4.32±0.10***	4.80±0.06***	4.60±0.11***	4.16±0.07***

*p<0.05, **p<0.01, ***p<0.001
